# Patterns of microbial diversity in three aquatic ecosystems of a Caribbean island

**DOI:** 10.1093/femsec/fiag031

**Published:** 2026-03-26

**Authors:** Vincent Hervé, Josie Lambourdière, Malika René-Trouillefou, Pascal Jean Lopez

**Affiliations:** Université Paris-Saclay, INRAE, AgroParisTech, UMR SayFood, 91120 Palaiseau, France; Laboratoire Biologie des Organismes et Ecosystèmes Aquatiques (BOREA), Centre National de la Recherche Scientifique, Muséum National d’Histoire Naturelle, Sorbonne Université, Institut de Recherche pour le Développement, Université des Antilles, 43 rue Cuvier 75005 Paris, France; Laboratoire Biologie des Organismes et Ecosystèmes Aquatiques (BOREA), Centre National de la Recherche Scientifique, Muséum National d’Histoire Naturelle, Sorbonne Université, Institut de Recherche pour le Développement, Université des Antilles, 43 rue Cuvier 75005 Paris, France; Laboratoire Biologie des Organismes et Ecosystèmes Aquatiques (BOREA), Centre National de la Recherche Scientifique, Muséum National d’Histoire Naturelle, Sorbonne Université, Institut de Recherche pour le Développement, Université des Antilles, 43 rue Cuvier 75005 Paris, France

**Keywords:** eDNA, biomonitoring, biodiversity, community assembly, surface waters

## Abstract

The functioning of various aquatic ecosystems is greatly influenced by the composition of their microbial communities. However, the prokaryotic and eukaryotic organisms present in the microbiome remain to be characterized in the waters of various tropical islands. Here, we used DNA metabarcoding to assess differences in the richness and abundance of prokaryotic and eukaryotic microbial communities in coastal, mangrove, and urban surface waters in Guadeloupe (French West Indies). We found that turnover was an important driving force in these three compartments, and that the urban compartment was the most diverse. We identified 119 prokaryotic and 80 eukaryotic OTUs with differential abundance between these three compartments. Furthermore, functional predictions revealed the importance of photosynthetic organisms (including Bacillariophyceae, Chrysophyceae, Chlorophyceae, and Cyanobacteria) in the three compartments, and an enrichment of urban waters in chemoheterotrophic prokaryotes and eukaryotic consumers. Interestingly, we detected several putative harmful algal bloom taxa that had not yet been reported in Guadeloupe. By cataloging the taxa restricted to particular water bodies, this inventory will facilitate analyses of the long-term effects of urbanization and industrialization on the evolution of microbial assemblages in Guadeloupe.

## Teaser

Differences between urban, marine, and mangrove aquatic microbial communities in a Caribbean island.

## Introduction

The region encompassing the Caribbean Sea and the Gulf of Mexico is a hotspot of marine biodiversity, that is home to various endemic species, belonging to Teleostei and Mollusca (Gastropoda and Bivalvia) in particular (Miloslavich et al. 2010, Costello et al. 2017). However, Caribbean countries are facing major challenges such as urbanization and climate changes, that can have various impacts on water quality (Cashman et al. 2010). Both fresh and marine waters are affected because a large proportion of wastewater remains untreated. Moreover, the demand for water is increasing and there are problems related to water supply and sanitation (Cashman 2014, Fonseca-Salazar et al. 2016, Tosic et al. 2018, Gheuens et al. 2019) that are often due to low groundwater volumes. Lastly, environmental changes contribute to the emergence and outbreaks of new waterborne pathogens and the dispersal of antibiotic-resistant bacteria with potential consequences for human health (Edwards et al. 2017, Bastaraud et al. 2020). Studies describing and monitoring microbial diversity and species composition are therefore essential for the assessment of water quality (Azis et al. 2018), evaluations of restoration efforts, and to address the impact of anthropogenic factors on species diversity (Morin and McGrady-Steed 2004).

The aquatic diversity of bacteria and protists in the Caribbean region have been described, at various scales from ecoregion to single island, in marine waters (Miloslavich et al. 2010, Edgcomb et al. 2011, Miloslavich et al. 2011), freshwater environments (Cross et al. 2023), or along the fresh-marine water continuum (Campbell et al. 2015), but urban waters remain overlooked. Due to inadequate sewage treatment or the presence of domestic animals in the watershed, the microbial diversity of Caribbean coastal waters are also impacted by microbial contamination (Mukherjee et al. 2016, Badilla-Aguilar and Mora-Alvarado 2019, Serville-Tertullien et al. 2022, (Batantou Mabandza et al. 2024)). Another putative health concern due to aquatic micro-organisms in the Caribbean zone is the increase in the frequency and geographic extent of harmful algal blooms (HABs), and more specifically of ciguatera fish poisoning (CFP) (Cuellar-Martinez et al. 2018, Sunesen et al. 2021, Seymour and McLellan 2025). Finally, the recent increase in *Sargassum* influx into this zone may also bring new pathogens or exotic microbial taxa to Caribbean coasts (Hervé et al. 2021, Cox et al. 2025). Therefore, there is a need to catalog both prokaryotic and eukaryotic diversity across aquatic ecosystems in the Caribbean region (Valster et al. 2011, Laas et al. 2021, Härer et al. 2022) in order to develop relevant biomonitoring strategies (Michán et al. 2021).

National agencies and United Nations experts have revealed that the Guadeloupe archipelago (French West Indies) is facing urgent and important issues relating to water quality and availability (i.e. water scarcity, problem of sewage treatment, and industrial and agricultural pollution). Environmental DNA (eDNA) metabarcoding approaches have therefore been used to investigate in various Guadeloupean water bodies the microbial diversity of distinct taxonomic groups, such as bacteria from mangroves (Halary et al. 2024, Jamon-Haon et al. 2025), rivers and marine waters (Guyomard-Rabenirina et al. 2017, Mabandza et al. 2024), free-living amoebae (Delumeau et al. 2023, Vingataramin et al. 2024), or dinoflagellates in the coastal waters of both Guadeloupe and Martinique (Boisnoir et al. 2019). However, data about both prokaryotic and eukaryotic communities must be collected together to understand microbial assembly processes.

The objective of this study was to explore and compare the prokaryotic and eukaryotic diversity of surface waters in Guadeloupe through an eDNA metabarcoding approach. We sampled various bodies of water from cities, suburbs, industrial areas, and protected areas around Pointe-à-Pitre. This area is of particular interest in the context of the Human-Environment Observatory Littoral Caraïbe, which analyzes the consequences of anthropization for the socio-ecological system (Chenorkian 2024, Hervé et al. 2024). We collected 123 samples from marine, mangrove, and urban surface waters, and performed a metabarcoding survey to assess the diversity of the prokaryotic and eukaryotic communities. We hypothesized that due to anthropogenic activities, urban waters will harbor more microbial diversity than mangrove and marine waters. We also hypothesized that at the scale of the Guadeloupe Island, certain taxa might be shared between the three aquatic ecosystems. Lastly, we hypothesized that there are differences in taxonomic and functional group composition between these three ecosystems. Finally, this study also provides preliminary DNA-based information that can be relevant for long-term investigations of microbial diversity including putative waterborne pathogens.

## Materials and methods

### Studied area and sampling strategy

The Guadeloupe archipelago consists of several islands. Two of the largest islands, the volcanic island Basse-Terre and the limestone island Grande-Terre, are separated by a narrow channel known as Rivière Salée. The sampling area for this study corresponded to the city of Pointe-à-Pitre and the surrounding municipalities: Les Abymes and Le Gosier on Grande-Terre, and Baie-Mahault, Petit-Bourg and Goyave on Basse-Terre. The study area also includes the third largest commercial and industrial zone in France and its territories (Jarry/Baie-Mahault), the chief port and economic hub of Guadeloupe and the international airport (Supplementary Fig. S1). The sampling sites lay within a total sampling area of 127.5 (km^2^), 60.2% marine and 39.8% terrestrial area, with a maximum north-south distance between points of 23.55 km, and a maximum east-west distance between points of 10.62 km.

For mangrove, surface waters were collected along the Rivière Salée and the Grand Cul-de-sac Marin (a protected marine area). This last area corresponds to the largest mangrove in the Lesser Antilles. Samples from water bodies bordering mangroves (10 samples) were considered to be mangrove samples. We also collected some water samples from inland mangrove forests (4 samples) (Supplementary Fig. S2). The 44 marine samples were from coastal zones in the Grand Cul-de-Sac Marin and the Petit Cul-de-Sac Marin, with a typical distance from the shoreline at low tide of 1.1 km and a maximum distance of 4.7 km. The marine samples also include one marina (Bas du Fort) and three small fishing ports (9 samples) (Supplementary Fig. S3). The urban waters sampled were collected from open channels (33 samples), artificial ponds (4 samples), fountains (4 samples), street gutters (6 samples), and episodic rain puddles (9 samples) (Supplementary Fig. S4). These urban samples correspond to relatively stable (i.e. drainage ditches, artificial ponds) or temporary (i.e. street gutters, episodic rain puddles, city fountains) surface waters.

All the marine samples and most of the mangrove water samples were collected with a Niskin or Ruttner water sampler (OSIL, Germany, 1 l) at a depth of 1 or 2 m. If the water column was too short for sampling at this depth, samples were collected in a Falcon tube lowered to a depth of about 20 cm. Urban samples from very shallow sites (i.e. fountains, street gutters, episodic rain puddles) were collected by sucking the water up into a sterile pipette. The collected samples were kept on ice and in the dark until they arrived at the laboratory (less than 2 h). The filtration volume varies according to the kind of sample, but we usually filtered until the 25 mm filters of 0.22 µm pores (Isopore, Millipore) were saturated. For urban water samples, this corresponds to about 15 ml (from 5 to 50 ml), depending on the sample, and for marine and mangrove samples, to about 150 ml (from 100 to 200 ml). Information about the sampling sites is provided in Supplementary Table I. The filters were stored at −20°C until processing.

The surface waters analyzed in this study were collected in March (between the 15^th^ and 20^th^) and July (from the 11^th^ to 16^th^) 2017, corresponding to the so-called “dry” (45% of the samples) and “wet” seasons (55% of the samples), respectively. Noteworthy, Météo-France data revealed that cumulative rain levels in the two periods were about the same, of the order of 2.5 mm/day, and that daily temperatures were also similar, at ∼26°C and 28.5°C, respectively.

### DNA extraction and sequencing

Environmental DNA was extracted from the filters with the DNeasy PowerBiofilm Kit (Qiagen), in accordance with the manufacturer’s recommendations. Total DNA was then quantified with Quant-it PicoGreen (Invitrogen, Thermo Fisher Scientific Carlsbad, CA, USA) and a CFX96 Real-Time System, C1000 Touch Thermal Cycler (Biorad, California, USA). DNA libraries were prepared with universal primers targeting both prokaryotes and eukaryotes. We amplified the V4-V5 region of the 16S rRNA with the 16S-515F-Y (5′-GTGYCAGCMGCCGCGGTAA-3′), and 16S-926R (5′-CCGYCAATTYMTTTRAGTTT-3′) primers, which bind to both bacterial and archaeal sequences (Walters et al. 2016). For amplification of the V4 region of the 18S rRNA gene, we used the TAReuk454FWD1 (5′-CCAGCASCYGCGGTAATTCC-3′) and TAReukREV3 (5′-ACTTTCGTTCTTGATYRA-3′) (Stoeck et al. 2010) primers. For each sample, we duplicated the initial DNA amplification in a final volume of 25 μl in accordance with the instructions supplied with the Taq Q5 Hot Start High Fidelity DNA Polymerase (New England Biolabs). The PCR products were checked by electrophoresis on agarose gels, purified with Agencourt AMPure XP beads (Beckman Coulter), and quantified with a Qubit dsDNA HS assay kit (Thermo Fisher Scientific). We then quantified and pooled the technical replicates by sample. We generated a single library per amplicon type (16S or 18S) by pooling equal amounts of all samples for the amplicon type concerned. These libraries were prepared with 1 µg pooled DNA and the TruSeq Nano Library Preparation Kit (Illumina). We followed the manufacturer’s protocol except that we used a modified End-Repair mix to prevent the production of chimeric constructs, and the libraries were not finalized by a PCR cycle. The resulting libraries (16S or 18S) were quantified by qPCR and the paired-end (2 × 300 bp) sequencing was performed on a HiSeq 2500 by Fasteris SA (Plan-les-Ouates, Switzerland).

### Taxonomic profiling based on the 16S and 18S rRNA gene amplicons

The two amplicon sequences were analyzed independently with *mothur* software version 1.39.5 (Schloss et al. 2009), as previously described (Hervé et al. 2021). First, contigs between read pairs were assembled. Barcode and primer sequences and low-quality sequences were then removed (minimum length of 330 bp and maximum length of 410 bp, with the removal of any sequence with ambiguous bases or with homopolymers longer than 8 bp). The sequences were aligned with the SILVA SSU reference database v138 (Quast et al. 2013) and preclustered (pre.cluster, diffs = 1). Singletons were excluded, and chimeras were removed with *vsearch* (Rognes et al. 2016) implemented in *mothur*. Sequences were classified with the naive Bayesian classifier (Wang et al. 2007) implemented in *mothur* with the SILVA reference database release 138 and the PR2 database version 4.12 (Guillou et al. 2013) for the 16S and 18S rRNA gene reads, respectively. After classification, non-prokaryotic, chloroplast, mitochondrial (for the 16S rRNA gene dataset), non-eukaryotic (for the 18S rRNA gene dataset), and unknown (for both 16S and 18S rRNA amplicons) sequences were excluded. We accounted for differences in sampling effort by randomly sampling 20 968 and 28 616 sequences from the 16S and 18S rRNA gene datasets, respectively, from each sample with *mothur*. This gave a sequencing depth sufficient to cover most of the diversity within each sample (Supplementary Fig. S5). Finally, operational taxonomic units (OTUs) were generated with the *vsearch* distance-based greedy clustering algorithm, with an OTU defined at the 97% and 99% sequence similarity levels for 16S and 18S rRNA gene reads, respectively. The raw sequence data have been deposited in the NCBI Sequence Read Archive under BioProject PRJNA1285926.

### Diversity and statistical analyses

Rarefaction curves were generated with *mothur*. Statistical analyses were performed with R software version 4.3.1. Data were manipulated and visualized with the *tidyverse* collection of R packages (Wickham et al. 2019). *Alpha* and *gamma* diversities were measured by calculating Hill numbers (from q = 0 to q = 5) with the *hilldiv* package (Alberdi and Gilbert 2019). Kruskal–Wallis tests followed by post-hoc Benjamini–Hochberg correction were used to evaluate differences in microbial richness. *Beta* diversity was visualized by principal coordinate analyses (PCoA), constructed from Bray–Curtis distances calculated with *mothur* (Schloss 2008). Changes in microbial community structure were investigated by analysis of similarities (ANOSIM) implemented in *mothur* and by non-parametric permutational multivariate analysis of variance (PERMANOVA) implemented in the *vegan* function *adonis* (Oksanen et al. 2015). We evaluated the relationship between spatial distance and microbial community composition by performing Mantel tests with the *ecodist* package (Goslee and Urban 2007), based on Pearson correlations, with 10^6^ permutations, using Bray–Curtis distances for the microbial communities and Euclidean distances for the spatial matrix. The partitioning of *beta* diversity was performed with the *betapart* package and the Jaccard family index (Baselga and Orme 2012). For the identification of OTUs with differential abundances between ecosystems, we used the LEfSe algorithm implemented in *mothur*, retaining only OTUs with a logarithmic LDA score > 3 and *P* < 0.05 (Segata et al. 2011).

### Functional inference of microbial OTUs

Several databases that have classified aquatic protists by trophic mode are available (Schneider et al. 2020, Singer et al. 2021, Mitra et al. 2023). We chose to use the classification proposed by Singer et al., which assigns protists to three major trophic modes: phototrophic, parasitic and consumers. Eukaryotic taxa were considered photosynthetic if their cells were reported to possess chloroplasts (Decelle et al. 2015). The OTUs considered here to be phototrophs corresponded to Alveolata (Colpodellidea and Dinoflagellata), Archaeplastida (all OTUs), Hacrobia (Haptophyta and Cryptophyta), Stramenopiles (Bacillariophyta, Bollidophyceae, Chrysomerophyceae, Chrysophyceae, Dictyophyceae, Eustimatophyceae, Pelagophyceae, Phaedophyceae, Pinguilophyceae, Raphidophyceae, and Xanthophyceae), and Rhizaria (Chlorarachniophyceae).

For bacteria and archaea, potential function was predicted with the Functional Annotation of Prokaryotic Taxa (FAPROTAX v1.2.6), which was initially designed for marine samples (Louca et al. 2016) and has been shown to be useful for terrestrial ecosystems, and for human and animal microbiomes (Sansupa et al. 2021). The predicted functional roles were visualized with the *pheatmap* R package, using Euclidean distances and the complete linkage clustering method.

### Putative harmful algal taxa

The species implicated in harmful algal bloom (HAB) events are listed in various databases including the Harmful Algal Event Database (HAEDAT), ocean biodiversity observation system (OBIS), and the IOC-UNESCO Taxonomic Reference List of Harmful Microalgae (IOC-UNESCO databases; https://www.marinespecies.org/hab/). This IOC-UNESCO taxonomic reference list includes 31 diatoms, 8 haptophytes, 82 dinoflagellates, 7 raphidophyceans, 3 dictyochophyceans, 43 cyanobacteria, and 4 species assigned to a gray list. We search our dataset for the presence of OTUs that could be assigned, at genus level, to putative HAB taxa listed in the IOC-UNESCO database (Supplementary Table II).

## Results

### Diversity patterns of the microbial communities

We analyzed 123 DNA samples from surface water collected in mangrove (*n* = 14), coastal marine (*n* = 53), and urban (*n* = 56) environments (Supplementary Fig. S1). After quality filtering and subsampling steps, we obtained a total of 24,073 prokaryotic and 22,864 eukaryotic OTUs. Rarefaction curves showed that the communities were sufficiently well sampled for investigations of microbial diversity patterns in our samples (Supplementary Fig. S5).

**Figure 1:**
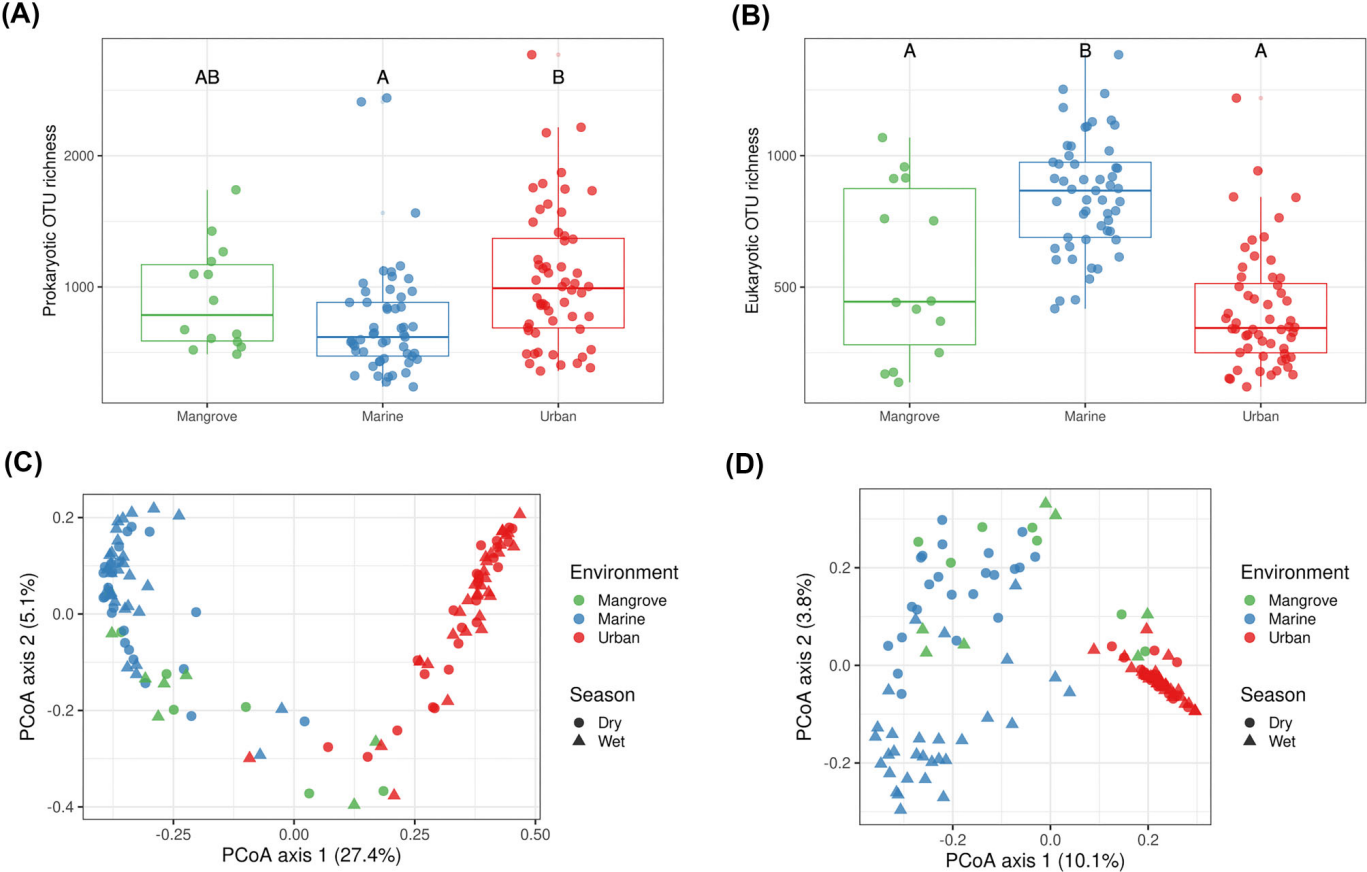
*Alpha* and *beta* diversity. *Alpha* diversity indices of surface water microbial communities.(A) observed prokaryotic OTU richness and (B) observed eukaryotic OTU richness. To compare the alpha diversity indices between compartments, we used Kruskal–Wallis test followed by Dunn’s test with Benjamini–Hochberg adjustment. Principal coordinate analyses (PCoA) of the microbial community composition based on Bray–Curtis dissimilarity matrices showing differences in community composition between surface water samples. (C) Prokaryotic community, and (D) Eukaryotic community. The samples were collected during the dry and wet season.

Prokaryotic richness in urban areas was significantly higher than that in marine samples (*P* < 0.001) but not than in mangrove samples (Fig. 1A). For eukaryotes, marine samples were significantly richer than mangrove and urban samples (*P* < 0.001) (Fig. 1B). These analyses were refined by the calculation of Hill’s numbers. The *alpha* diversity profiles obtained revealed that, for prokaryotes, the most diverse community was that of urban surface waters if all OTUs were considered equally (OTU richness, q = 0) whereas that of mangrove waters was the most diverse if the analysis focused on the most dominant OTUs (dominant OTUs, q = 2 and above) (Supplementary Fig. S6). The *alpha* diversity profiles of eukaryote communities also differed according to the ecosystem and the diversity order considered. Richness was highest (q = 0) for the marine communities and lowest for the urban communities (Supplementary Fig. S6). If only the most dominant OTUs were considered (q = 5), the mangrove communities had the lowest levels of diversity. We found no difference in prokaryotic richness between the seasons (*P* = 0.50) whereas eukaryotic richness was higher in the wet season than in the dry season (*P* = 0.039).

A principal coordinates analysis (PCoA) based on the Bray–Curtis distances of the prokaryotic communities from the three ecosystems (i.e. mangrove, marine, and urban surface waters) showed no significant differences (PERMANOVA, R² = 0.010, *P* = 0.199) between samples collected in the two seasons (Fig. 1C–D). For eukaryotes, season had a weak but significant effect on community composition, as highlighted by the low proportion of the explained variance (PERMANOVA, R² = 0.018, *P* < 0.05). We did not therefore take season into account in subsequent analyses. We found that the communities from marine, mangrove, and urban samples differed significantly for both prokaryotes (ANOSIM, *R* = 0.70, *P <* 0.0001; PERMANOVA, R² = 0.314, *P* < 0.001) and eukaryotes (ANOSIM, *R* = 0.72, *P <* 0.0001; PERMANOVA, R² = 0.116, *P* < 0.001) (Fig. 1C–D). A PCoA based on Jaccard distances yielded similar patterns (Supplementary Fig. S7). Furthermore, if we considered only urban environments, we found that samples tended to cluster according to their origin (i.e. open channel, fountain, artificial pond, street gutter or episodic rain puddle) for both prokaryotic and eukaryotic communities (Supplementary Fig. S8).

To find out if there was a distance-decay relationship, we examined the relationship between spatial distance and the composition of the microbial community. Considering all samples, we found no distance-decay relationship for prokaryotic or eukaryotic communities across ecosystems (Mantel test, *P* > 0.05, Supplementary Fig. S9). However, within ecosystems, a significant distance-decay relationship was observed for both prokaryotes and eukaryotes in the mangrove and marine ecosystems (Mantel test, *P* < 0.05) but not in the urban ecosystem (Supplementary Fig. S9).

**Figure 2:**
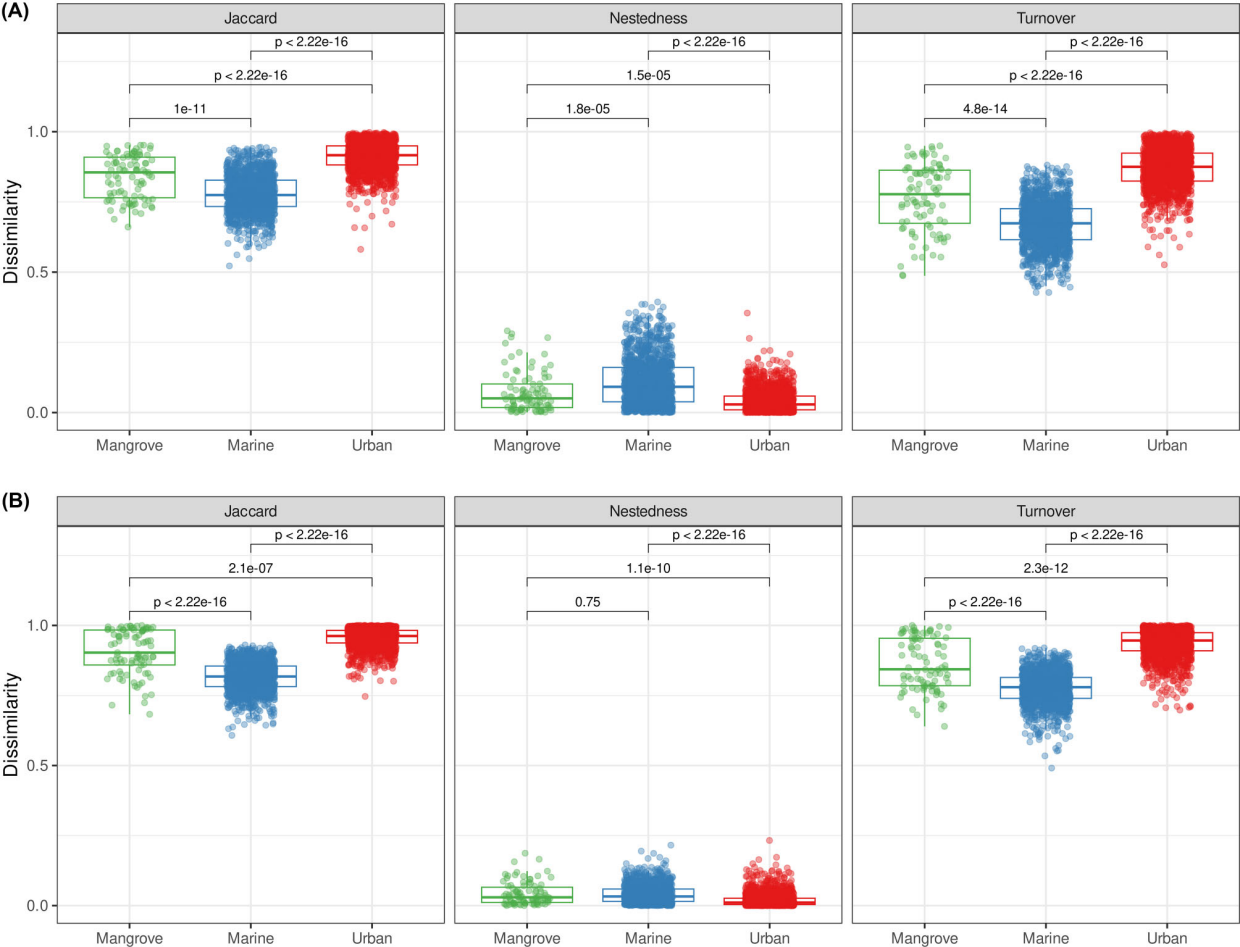
*Beta* diversity partitioning of each surface-water environment. The values correspond to the Jaccard, nestedness and turnover components for(A) prokaryotes and (B) eukaryotes. Significance of the differences between the environments was tested with Kruskal–Wallis test followed by Wilcoxon tests.

We further investigated the differences in OTU composition between sites by studying *beta* diversity patterns through analyses of turnover and nestedness components (Fig. 2). These analyses revealed that, for both prokaryotes and eukaryotes, the Jaccard dissimilarity index differed significantly between the three compartments (*P* < 0.001) and that this index was highest in urban environments, followed by mangrove and marine environments. Turnover (i.e. OTU replacement) predominantly drove *beta* diversity (median values for prokaryotes: 94.1%, 87.8%, and 96.8% for mangrove, marine, and urban waters, respectively; median values for eukaryotes: 96.4%, 96.0%, and 98.9% for mangrove, marine, and urban waters, respectively). For both prokaryote and eukaryote microbial communities, turnover significantly differed between the three environments (*P* < 0.001). It was highest in urban areas followed by mangrove and marine environments (Fig. 2). Finally, nestedness was found to make a minor contribution (Fig. 2).

The *gamma* diversity profile analysis revealed that the overall OTU diversity was higher in urban surface waters than in the other two environments, for both prokaryotic and eukaryotic communities (Supplementary Fig. S10). Similar results were obtained with sample-based OTU accumulation curves (Supplementary Fig. S11). These results may partly reflect the diversity of samples in the urban environment, with some sites corresponding to permanent and others to temporary waters.

### Taxonomic distribution between surface waters

**Figure 3:**
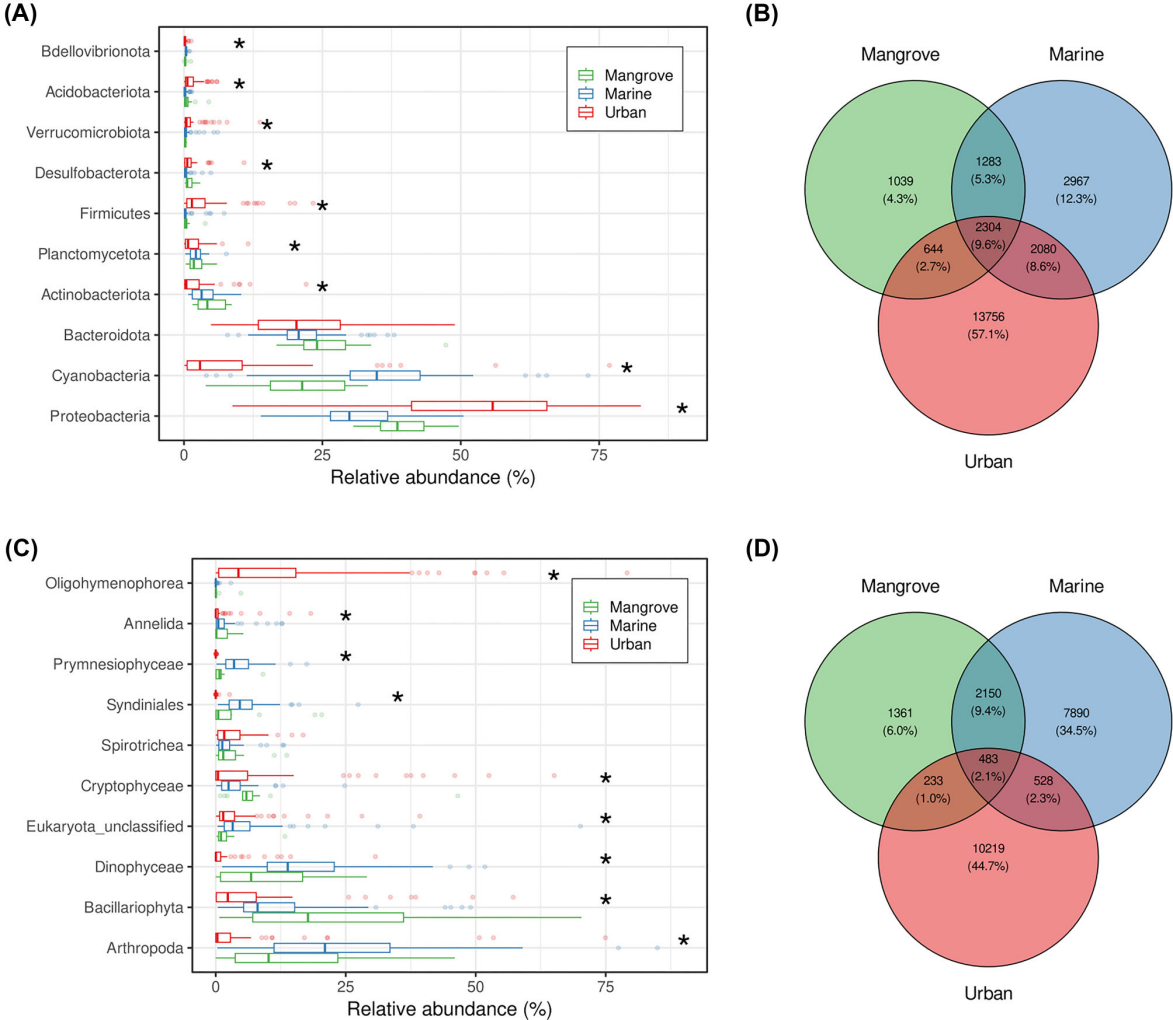
Difference in the relative abundances and shared OTUs in the three environments studied.(A) The boxplots illustrate the relative abundance of the 10 most abundant bacterial phyla in the three environmental compartments. Asterisk indicates a significant difference between these compartments (Kruskal–Wallis, *P* < 0.05). (B) Venn diagram of the shared bacterial OTUs; the OTU number and the corresponding percentage are indicated. (C) The boxplots illustrate the relative abundance of the 10 most abundant eukaryotic clades in the three environmental compartments. Asterisk indicates a significant difference between these compartments (Kruskal–Wallis, *P* < 0.05). (D) Venn diagram of the shared eukaryotic OTUs; the OTU number and the corresponding percentage are indicated.

In terms of OTU richness, prokaryote communities were dominated by *Proteobacteria* (accounting for a mean 24.6% of the OTUs), followed by *Bacteroidota* (20.7%), *Planctomycetota* (9.9%), and *Firmicutes* (6.1%) (Supplementary Fig. S12). However, relative abundance analysis revealed a different pattern, in which *Proteobacteria* were dominant (mean of 40.3%), followed by *Bacteroidota* (22.9%) and *Cyanobacteria* (21.8%) (Supplementary Fig. S12). Additional differences between compartments were found, with urban environments significantly enriched in *Proteobacteria, Firmicutes, Acidobacteriota*, and *Verrucomicrobiota* (Fig. 3A). Urban waters had the largest numbers of specific OTUs (57.1%) (Fig. 3B). The lowest percentage of distinct OTUs (4.3%) was found in mangroves, but this is likely due to the reduced sample size (Fig. 3B). Overall, 2,304 (9.6%) OTUs were found in at least one sample from each surface water category.

A comparison of eukaryotic communities showed more pronounced differences in OTU composition between the three surface water environments, particularly for urban areas. In terms of OTU richness, mangrove and marine waters were dominated (mean of 32.0% within these two compartments) by members of the superphylum Alveolata (with Dinoflagellata accounting for 83.8% and 80.5% of OTUs for marine and mangrove waters, respectively), followed by Opisthokonta (15.4%), Stramenopiles (14.4%), and Hacrobia (8.9%) (Supplementary Fig. S13). Urban waters were dominated by Opisthokonta (22.2%), followed by Alveolata (14.4%), Stramenopiles (15.8%), Archaeplastidia (13.4%), and Rhizaria (10.0%) (Supplementary Fig. S13). In terms of relative abundance, the dominant groups in mangroves were Stramenopiles (23.4%), Opisthokonta (23.0%), Archaeplastida (22.5%), and Alveolata (17.8%). Marine waters were dominated by Opisthokonta (35.8%), Alveolata (26.3%), and Stramenopiles (13.8%). Urban waters were dominated by Alveolata (25.2%), Opisthokonta (23.5%), Archaeplastida (17.9%), and Stramenopiles (14.2%) (Supplementary Fig. S13). Interestingly, in urban waters Oligohymenophorea (Phylum Ciliophora) was the most abundant class, on average, among Alveolata (52.4%), but with an abundance that varied considerably between urban samples (Fig. 3C, Supplementary Fig. S13). An analysis of the relative abundances of various eukaryotic clades revealed differences between the three compartments, particularly for Dinophyceae, Bacillariophyta, and Arthropoda (Fig. 3C). The OTUs common to these compartments accounted for only 2.1% of total richness (Fig. 3D), demonstrating clear differences in the composition of eukaryotic communities between aquatic compartments. Again, the number of specific OTUs was highest in urban waters (44.7%) (Fig. 3D).

### Identification of environment-specific biomarkers

**Figure 4:**
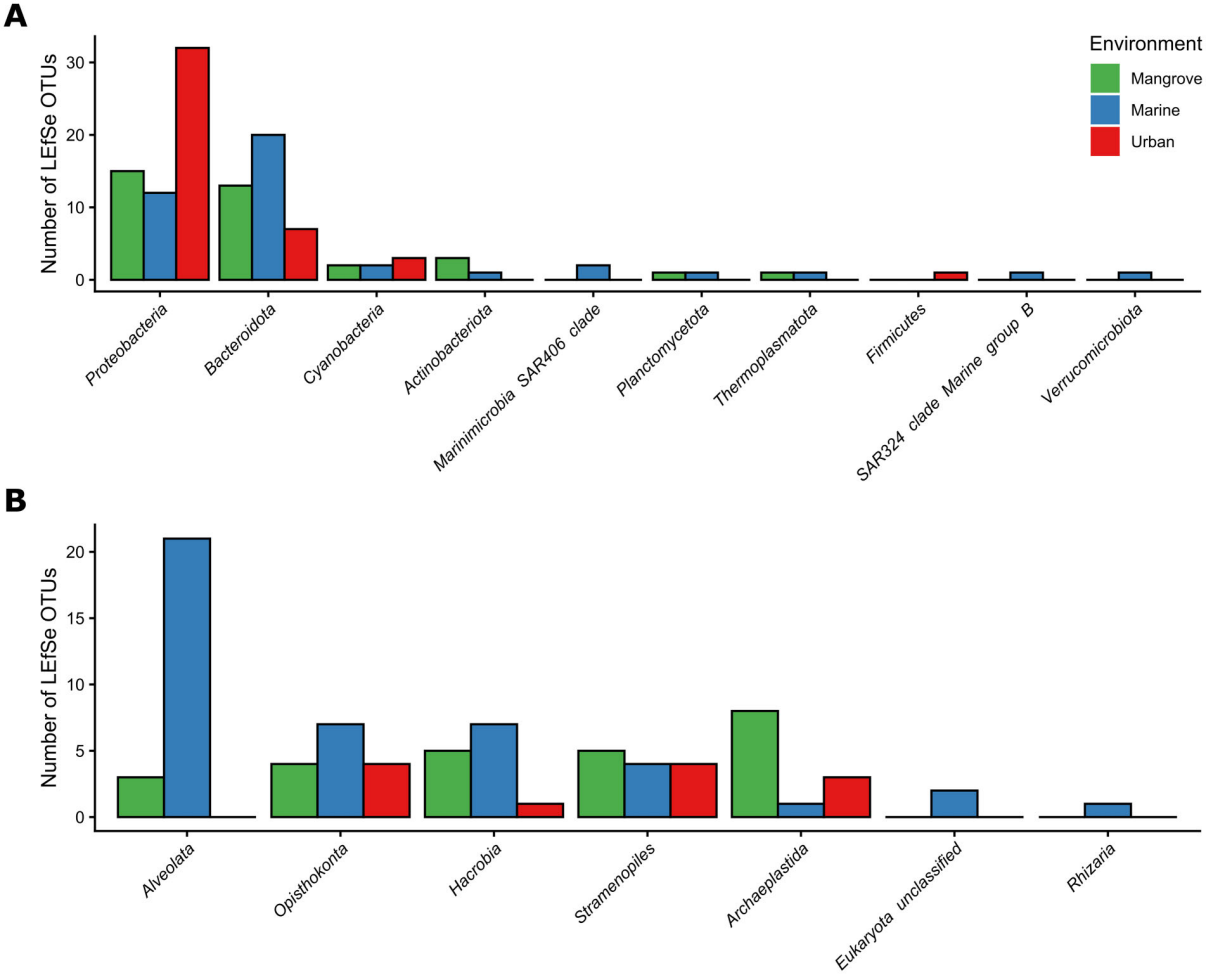
Taxonomic distribution of the environment-specific OTUs identified by LEfSe analysis. (A) prokaryotes and (B) eukaryotes. The bar color corresponds to the compartment where OTUs are differentially abundant.

LEfSe analysis showed that 119 OTUs, corresponding to 10 prokaryotic phyla, had significantly different abundances between the three compartments (Fig. 4A and Supplementary Table II). These OTUs included 29 corresponding to order *Flavobacteriales* for which a significant enrichment was observed in marine (17 OTUs) and mangrove (9 OTUs) waters, including members of the NS2, NS4 and NS5 marine groups. *Burkholderiales* (*Proteobacteria*) was highly represented in urban environments (17 OTUs), including members of the *Comamonadaceae* family and genus C39. We also found *Cyanobacteria* OTUs specific to each of the three compartments.

Differential abundance analysis also identified 80 eukaryotic OTUs corresponding to eight Supergroups (PR2 Rank 2) (Fig. 4B and Supplementary Table II). A significant enrichment in the order Cryptomonales (Cryptophyceae), the families Maxillopoda (Crustacea), and polar centric Mediophyceae (Bacillariophyta) was observed in marine and mangrove environments, whereas an enrichment in unclassified Dinophyceae was observed in marine environments. An enrichment in Cryptomycotina (Fungi) was observed only in urban environments. We identified eight OTUs from division Chlorophyta (PR2 Rank 3) displaying enrichment in the mangrove compartment.

### Putative functional roles of prokaryotic communities

**Figure 5:**
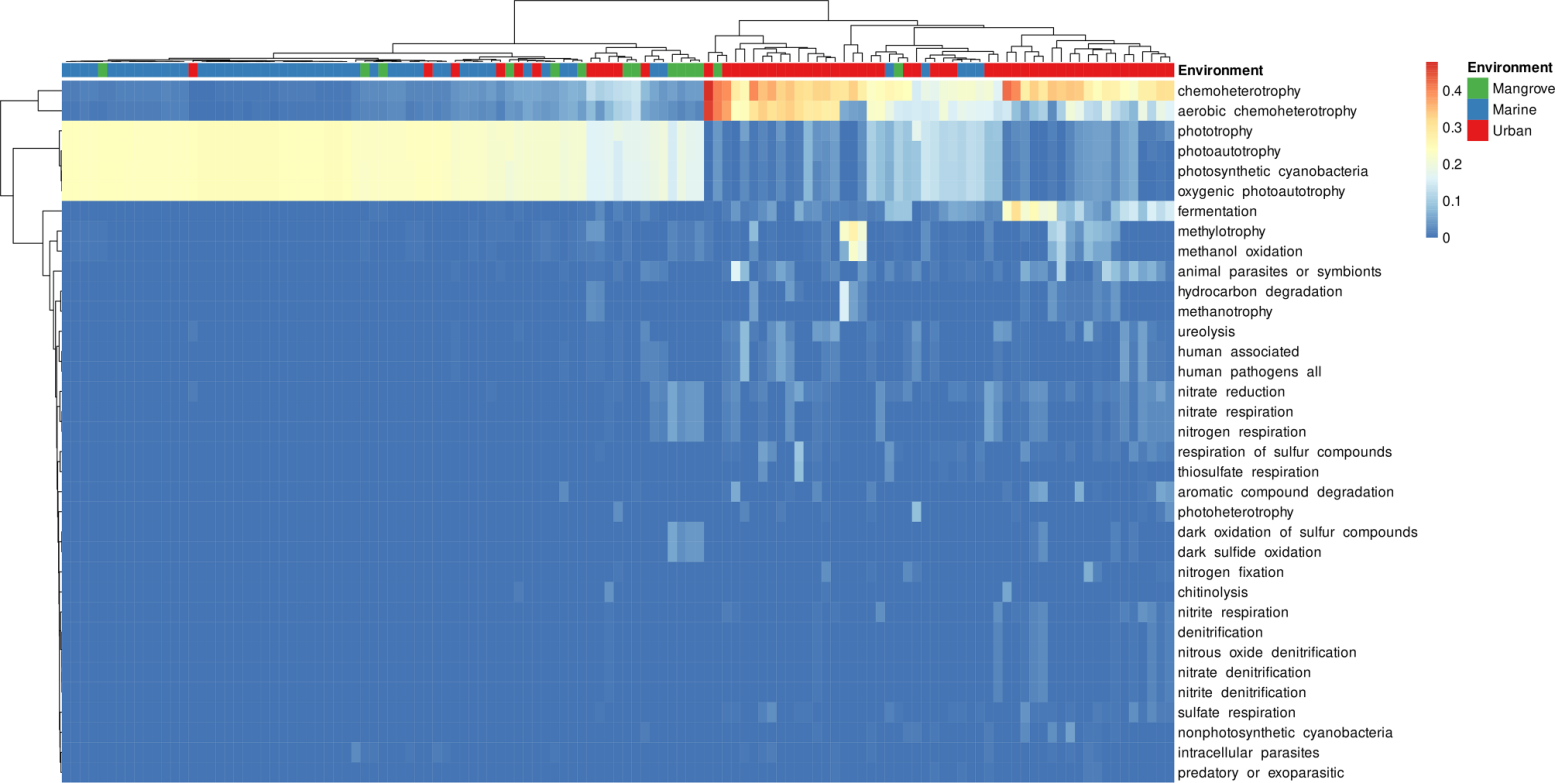
Heatmap of predicted functions based on the FAPROTAX database. The 35 categories presented here correspond to 3,815 prokaryotic OTUs. Functions were clustered with the weighted pair group method centroid (WPGMC) algorithm based on Euclidean distances. The color scale represents the proportion of each predicted function within a sample.

We investigated the putative functions of prokaryotes using FAPROTAX. We identified 3,815 prokaryotic OTUs (15.8% of the global dataset, corresponding to 39% of the total relative abundance) that could be functionally assigned to 61 functional groups with at least one record (not shown). The most diverse of these functional groups corresponded to chemoheterotrophy (8.6% and 13.6% of overall OTU richness and abundance, respectively), phototrophy (2.6% and 20.4%), fermentation (2.1% and 2.9%), and intracellular parasites (1.7% and 0.27%) (Fig. 5). These data demonstrate the high abundance of primary producers in surface waters, especially in marine and mangrove environments. By contrast, chemoheretotrophs were more abundant in urban surface waters (Fig. 5).

In total, 615 prokaryotic OTUs were classified as phototrophs. Class *Cyanobacteria* (575 OTUs) was the most diverse group identified, with some highly abundant OTUs. In marine waters, the most abundant *Cyanobacteria* OTU (Otu00001) was assigned to the genus *Synechococcus*, accounting for 31.4% of the total abundance in this compartment. This *Synechococcus-*related OTU was also dominant in mangrove samples (11.4% of the total abundance within this compartment), but accounted for only 0.06% of total abundance in urban samples. The two most abundant *Cyanobacteria* OTUs in urban waters (Otu00009 and Otu00004) were assigned to the genus *Cyanobium* PCC-6307 (together these two OTUs accounted for 4.11% of the total abundance within this compartment). In total, 425 *Cyanobacteria* OTUs were found only in this compartment, and only 25 OTUs were common to the other compartments. Our findings show that the diversity of photosynthetic *Cyanobacteria* varies significantly between brackish, marine and urban waters.

### Trophic status of eukaryotic surface-water communities

**Figure 6:**
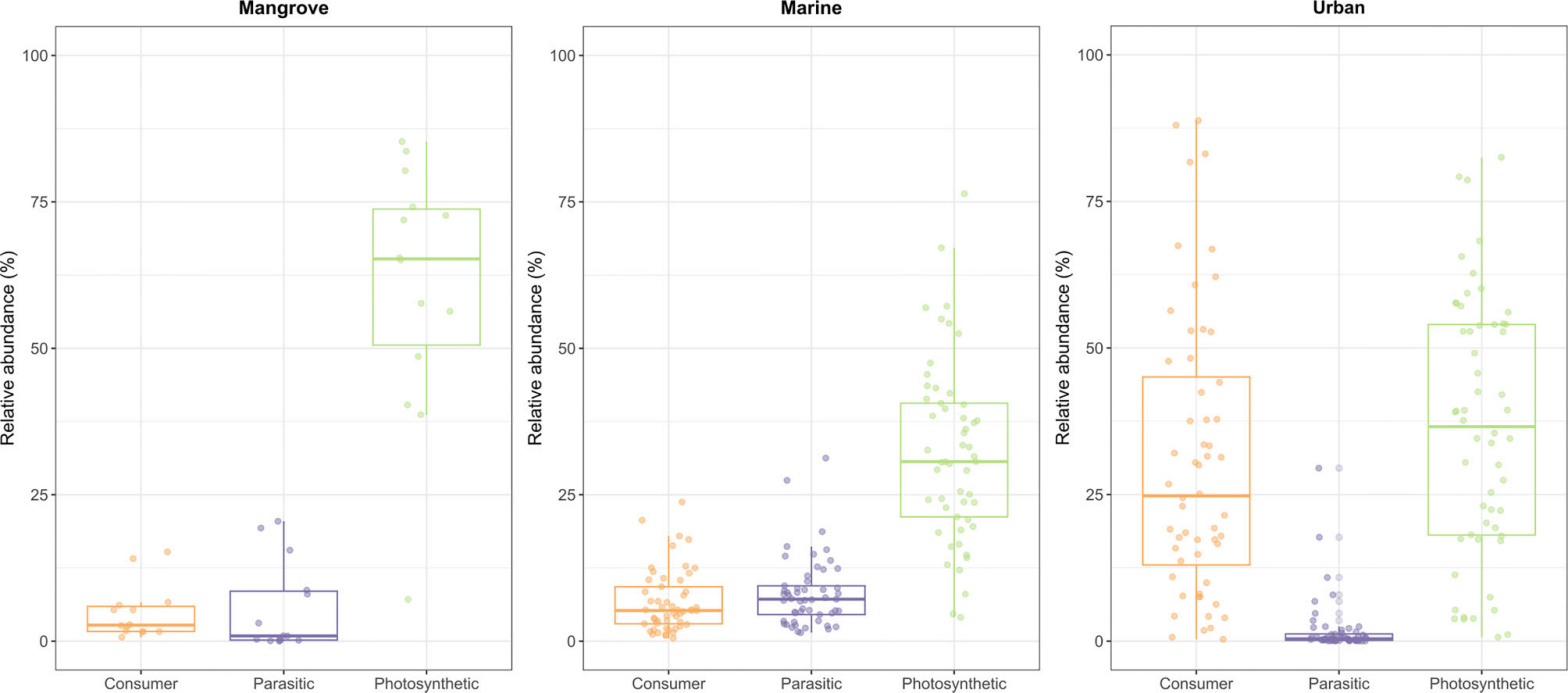
Relative abundance of eukaryotic OTUs assigned to three major trophic modes: phototrophic, parasitic and consumers. The data correspond to the relative abundance per site of the trophic groups and in each compartment.

Based on PR2 assignment, we were able to associate functional groups with 60.7% of the eukaryotic OTUs from mangrove, 52.6% of those from marine environments, and 57.8% of those from urban environments. Phototrophs were the most abundant in all three environments, and the consumer group was particularly abundant in urban environments (Fig. 6). We refined our analysis by considering only the most abundant OTUs, defined here as those with a relative abundance above 1% in at least one of the three compartments. We identified 17 classes of abundant OTUs with a single trophic status: five classes of Alveolata, six Archaeplastidia, two Stramenopiles, two Hacrobia, and two Rhizaria. We found that Bacillariophyta were the most abundant phototrophic organisms (22.3%, 12.4%, and 7.8% of total abundance in the mangrove, marine, and urban compartments, respectively). Mamiellophyceae displayed differential abundance, accounting for 13.9% of total abundance in mangrove waters but only about 4.0% in marine waters and 0.1% in urban waters. Prymnesiophyceae was significantly more abundant in the marine compartment than the other compartments (∼4.7% of total abundance in marine waters versus 1.2% in mangrove and 0.01% in urban waters), and Chlorophyceae was more abundant in the urban compartment (∼12.3% of total abundance in urban waters versus 0.04% in mangrove and 0.02% in marine waters) (Supplementary Fig. S14). Urban surface waters presented a much larger abundance of five classes of consumers (Oligohymenophorea, Phyllopharyngea, Endomyxa, Filosa-Sarcomonadea, and Bicoecea) (Supplementary Fig. S14). For parasitic OTUs, class Colpodellidea was present mostly in urban areas, and class Syndiniales, a ubiquitous group of protist parasites, was present mostly in marine and mangrove waters (Supplementary Fig. S14).

### Putative harmful algae

**Figure 7:**
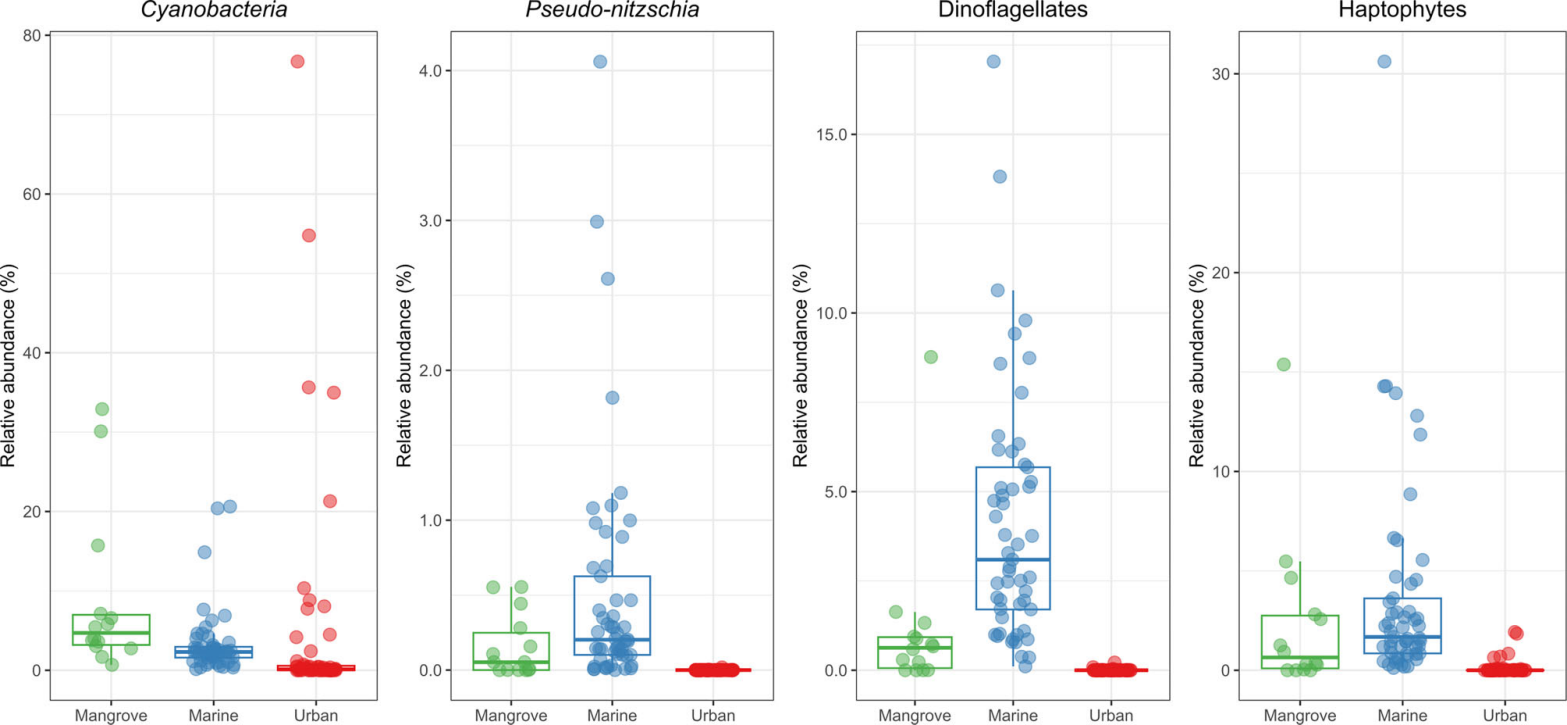
Relative abundance per site of selected groups of putative harmful algae in mangrove, marine and urban surface waters. Note that the scale of the relative abundance depends on the putative HAB considered.

Based on the IOC-UNESCO taxonomic reference list of harmful microalgae, we identified 1,207 eukaryotic OTUs corresponding to genera implicated in HAB events (Supplementary Table II). In particular, we identified 42 OTUs assigned to genus *Pseudo-nitzschia*, 15 *Alexandrium* OTUs (including one *Alexandrium affine* and one *Alexandrium andersonii*), 445 *Pyrodinium* OTUs (including 389 *Pyrodinium bahamens* OTUs), 32 *Prorocentrum* OTUs (including one *Prorocentrum emarginatum* and one *P. cassubicum*), 324 *Chrysochromulina*, and 43 *Amphidinium* OTUs (including OTUs assigned to the species *Amphidinium klebsii, A. gibbosum*, and *A. carterae*). Using the list of *Cyanobacteria*, we also identified 45 OTUs corresponding to the genera *Annamia, Calothrix, Cyanobium, Cylindrospermum, Lyngbya, Microcystis, Phormidium, Planktothrix*, and *Snowella* (Supplementary Table II). A further analysis of some of these genera revealed a heterogeneous distribution, depending on compartment, with some sites presenting a much higher abundance (Fig. 7).

## Discussion

The primary goal of this study was to describe and compare multiple microbial groups within and between various bodies of surface water in Guadeloupe, a French overseas territory located in the Lesser Antilles. We evaluated the overall richness and dominant microbial groups within a geographic area encompassing the most densely populated and urbanized areas of Guadeloupe and adjacent areas, including a protected marine area (Supplementary Fig. S1). This area is also of particular interest to the Caribbean Coast Human-Environment Observatory, which investigates socio-environmental trajectories (Hervé et al. 2024).

*Alpha* diversity profiles showed that urban water samples presented the highest prokaryotic diversity but the lowest eukaryotic diversity, along with a high turnover. High turnover was also found in mangrove and marine environments. These results were unexpected because many aquatic protists have a more passive mode of dispersal than observed in other habitat groups (Pettersen et al. 2022). Therefore our findings probably imply that local biological mechanisms, such as environmental selection and the relative isolation of habitats, may be the main driving forces behind the high degree of OTU replacement (Lawton 1999, Soininen 2010, Lindström and Östman 2011, Langenheder and Lindström 2019). In addition, a relative isolation of habitats was also expected in this study, due to the major differences between the types of urban water sampled.

We identified 24,073 prokaryotic and 22,864 eukaryotic OTUs, which correspond to our knowledge to the largest number of taxa ever described in a single study for this French overseas territory. Among them, 2,304 prokaryotic OTUs (9.6% of the total number of OTUs) were common to all three compartments, whereas this was the case for only 483 eukaryotic OTUs (2.1%). The existence of common taxa to different aquatic ecosystems may be explained by the presence of generalist taxa and/or a high dispersal rate (James et al. 2024, Rain-Franco et al. 2024). The lower number of shared eukaryotic OTUs suggest a high divergence in diversity composition and, probably, a higher number of specialized eukaryotes. It’s interesting to note that several diatom genera, including *Chaetoceros, Cyclotella, Skeletonema*, and *Thalassiosira*, were among the most prevalent eukaryotic OTUs shared by the three compartments. These genera are known to be able to colonize a wide range of habitats (Tison-Rosebery et al. 2017, Piredda et al. 2018, Pierella Karlusich et al. 2025).

### Compositional differences between ecosystems and putative roles of bacterial communities

We found a number of significant differences in the relative abundance of several bacterial phyla between compartments (Fig. 3). Among them, we found planktonic taxa characteristic of the marine environment, such as the SAR11 clade (*Alphaproteobacteria*), members of the NS marine groups (*Flavobacteriales*), and Marine Group II Archaea (*Thermoplasmata, Thermoplasmatota*), which are known to contribute to the global ocean carbon cycle (Alonso et al. 2007, Zhang et al. 2015, Giovannoni 2017). Interestingly, we also found that *Cyanobacteriia* were mostly abundant in coastal surface waters. Within this class, a single OTU assigned to *Synechococcus* CC9902 had a mean abundance of about 30% in marine samples. *Synechoccocus* is an abundant and widespread cyanobacterial genus in marine environments, but its abundance, physiological features, and gene expression are known to display spatial and temporal patterns of variation (Farrant et al. 2016, Dore et al. 2022, Li et al. 2023, Harding et al. 2025). In both freshwater and marine settings, it has been demonstrated that the release of nitrogenous compounds and, more broadly, eutrophication raise the concentrations of a number of cyanobacterial species, including *Synechococcus* (Rajaneesh et al. 2020, Wang et al. 2022, Wåhlström et al. 2024, Zuo et al. 2024). We therefore hypothesize that the high abundance of *Synechococcus* found in this study is a potential result of the eutrophication of Guadeloupean coastal waters which might be explained by the high levels of human and industrial activity in the study area, as well as the low overall efficacy of the local wastewater treatment plants.

Despite heterogeneity in the composition of the microbial communities of urban surface waters, this compartment consistently displayed a strong enrichment in *Proteobacteria* (Figs 3 and 4). The most abundant family was *Acetobacteraceae* (*Alphaproteobacteria*), which includes various taxa found in extreme environments, as well as acetous and acidophilic bacterial taxa that have been extensively studied for use in the food and industrial sectors (Guzman and Vilcinskas 2022, Degli Esposti et al. 2023). In this environment, the most abundant bacterial OTUs was assigned to *Roseomonas* genus, which includes acidophilic taxa (Rai et al. 2021, Guzman and Vilcinskas 2022) associated with human constructions due to their ability to establish biofilms within the concrete corrosion layer (Li et al. 2017). Urban samples were also enriched in *Burkholderiales* (*Gammaproteobacteria*), a large group of bacteria commonly found in soils and waters and which has been implicated in plant growth, nutrient cycling, and pollutant degradation (Coenye and Vandamme 2003). *Burkholderiales* also includes agents that are pathogenic in plants and animals, including humans (Voronina et al. 2015). The other abundant families were *Comamonadaceae, Rhodocyclaceae* (genus C39, and unclassified *Rhodocyclaceae*), and *Burkholderiaceae* (genus *Polynucleobacter*). Interestingly, genus C39 has been reported in bodies of freshwater, in association with mosquito larvae (Alfano et al. 2019). Another bacterial family that was common in urban samples is *Sporichthyaceae* (*Frankiales, Actinobacteria*) which have previously been reported in urban waters (Newton and McLellan 2015).

Predictions of function for the prokaryotic community revealed that the urban samples contained mostly chemoheterotrophs. The mangrove and marine surface waters predominantly contained phototrophs. The mangrove samples also presented functions related to nitrate and sulfur metabolism. This was expected, as mangrove sediments are known to play an active role in sulfur recycling (Mo et al. 2023) and because mangroves receive large amounts of nitrogen compounds from human activities (Nie et al. 2021, Mack et al. 2024). In urban environments, trophic status and many of the functions identified were related to fermentation, methylotrophy, hydrocarbon degradation, animal parasites, or symbionts. In particular, 171 OTUs (2.4% of the total number in this compartment) were assigned by FAPROTAX to the animal parasite or symbiont categories. The most abundant genera among these OTUs were *Roseomonas* (*Acetobacterales*) and *Prevotella* (*Bacteroidales*). These environmental bacteria have been found in the human microbiome and associated with infectious diseases in humans (Romano-Bertrand et al. 2016, Tett et al. 2021, Yeoh et al. 2022). Altogether our data reveal that there are several common bacteria in Guadeloupean urban and suburban areas that have been linked to humans and urban settings.

### Eukaryotic trophic group abundances in surface waters

Following to the classification proposed by Singer et al. (2021), phototrophic OTUs were the most abundant OTUs in mangrove samples. Eighteen of these OTUs were mangrove biomarkers, including the diatom genera *Chaetoceros, Conticribra, Cyclotella*, and *Thalassiosira*, and two OTUs assigned to the genus *Picochlorum* (Trebouxiophyceae, Chlorophyta). Chlorophytic phytoplankton are among the major contributors to aquatic primary production in a number of tropical and subtropical zones, but their abundance varies with period, season and location (Gaiser et al. 2004, Samanta and Bhadury 2018, Hilaluddin et al. 2020). We also identified five mangrove biomarkers classified as cryptophytes, including members of the genera *Hanusia* and *Plagioselmis*, which were most abundant in water samples from mangrove forests and the fringes of such forests, respectively. Although we did not examine phytoplankton communities along the salinity gradient, our findings are in line with a prior molecular study conducted on the island of Curaçao that revealed variations in diatom and green algae abundances between habitats, with diatoms being more prevalent in mangrove and salt ponds (Eckmann et al. 2023).

Marine surface waters were dominated by Dinophyceae but also contain abundant diatom genera including *Chaetoceros* (two biomarker OTUs), *Skeletonema, Thalassiosira*, and *Pseudo-nitzschia*. The most abundant diatom OTU was assigned to *Chaetoceros tenuissimus*, a cosmopolitan species of interest for analyses of water quality (Pastorino et al. 2022). In the urban environment, diatoms were the third most abundant class of phototrophic organisms after chlorophytes and the cryptophytes. In this environment, two other abundant diatoms were *Nitzschia palea* and *Cyclotella meneghiniana*, that are common freshwater species previously described in water quality assessments in the French West Indies (Heinry et al. 2024). Our data confirmed the importance of investigating diatoms and other photosynthetic algae when analyzing the quality of temporary or permanent salt and fresh waters, with potential implications for the understanding of their ecological roles in tropical aquatic ecosystems (Chen et al. 2016, Samanta and Bhadury 2018, Taurozzi et al. 2024).

Consumer taxa were found in marine and mangrove surface waters, but this trophic mode was most abundant in urban samples (Fig. 6). Among them, the class Oligohymenophorea (Ciliophora, Alveolata) accounted for 36.5% of the relative read abundance in this compartment. Our data confirm previous studies reporting ciliates to be among the most abundant groups in freshwater ecosystems (see Liu et al. 2022 and associated references). Oligohymenophorea species are often used in wastewater treatment (Muñoz-Palazon et al. 2019), and species such as *Paramecium multimicronucleatum* and *Vorticella microstoma* have been shown to be useful for the bioremediation of heavy metal contamination (Liaqat et al. 2022, Zahra et al. 2023), or to survive in contaminated industrial effluent (Rehman et al. 2007). Oligohymenophorea are major contributors to the microbial loop, preying on bacteria and small protists (Carvalho da Silva and Fernandes 2023, Abraham et al. 2024). Our findings indicate that Oligohymenophorea may actively contribute to the microbial composition in Guadeloupe’s surface waters, despite the observed variations in both richness and abundance between sites.

### Putative toxic or harmful taxa

As in other regions of the world, HAB have been documented in Latin America and the Caribbean (LAC), with ecological, social, and economic consequences (Cuellar-Martinez et al. 2018, Hallegraeff et al. 2021, Sunesen et al. 2021, Ayache et al. 2023, Igwaran et al. 2024). Using the list of genera provided by the IOC-UNESCO dataset, we investigated the presence of putative HAB micro-organisms in Guadeloupe. We identified 1,207 eukaryotic OTUs and 31 prokaryotic OTUs corresponding to putative HABs. Even if we did not isolate strains nor measure any toxin levels, we found relevant to report several of these taxa that present various ecological roles and that were not often described in the French West Indies.

In particular, we identified OTUs belonging to *Cyanobacteria* (nine genera: *Annamia, Calothrix, Cyanobium, Cylindrospermum, Lyngbya, Microcystis, Phormidium, Planktothrix*, and *Snowella*) that include putative toxic but also non-toxic taxa. *Cyanobium* which was the most abundant genus in all three water compartments, is commonly found in both freshwater and marine ecosystems and can produce cyanotoxins (Jakubowska and Szelag-Wasielewska 2015, Chorus and Welker 2021, Dirks et al. 2024). Interestingly, among the environment-specific biomarkers identified here, we found two *Cyanobium* OTUs among the mangrove biomarkers and one among the urban biomarkers. *Planktothrix*-related OTUs were present in 64% of the urban samples and in one marine sample but were absent from mangrove. These findings are in contrast to earlier data for this species in Guadeloupe’s marine mangroves (Guidi-Rontani et al. 2014). However, this earlier study concentrated on mat sediment samples, whereas we examined water samples. Altogether, our molecular investigations reveal the presence of potentially harmful cyanobacteria in various aquatic environments, which should encourage stakeholders to establish monitoring systems and carry out toxicity assessments for this bacterial phylum.

For eukaryotes, we focused on the genus *Pseudo-nitzschia* because the incidence of this potentially toxic diatom has increased globally over the last two decades, including in the Caribbean region (Liefer et al. 2013). We identified 42 OTUs assigned to *Pseudo-nitzschia* that were present in marine and mangrove samples. These data suggest that more detailed analyses of the presence of this potentially harmful genus in Guadeloupean waters are required even if it should be borne in mind that not all species are harmful. Indeed, the production of domoic acid (DA), in which causes amnesic shellfish poisoning (ASP), greatly depends on the species and ecotype of *Pseudo-nitzschia*, cell physiology and environmental parameters (Santiago-Morales and García-Mendoza 2011, Lema et al. 2017, Sauvey et al. 2023, He et al. 2024).

Dinophyceae capable of producing paralytic shellfish toxins are another major concern in the wider Caribbean area (Landsberg et al. 2006, Usup et al. 2012, Morquecho 2019, Sunesen et al. 2021, Arteaga-Sogamoso et al. 2022, Núñez-Vázquez et al. 2022). We identified several OTUs assigned to some of the well-documented HAB species, such as *Alexandrium affine, Alexandrium andersonii, Prorocentrum emarginatum, Prorocentrum cassubicum*, and *Pyrodinium bahamense*. Moreover, our findings of the presence of potentially harmful dinoflagellates in the marine pelagic zone are complementary to those of previous studies on benthic dinoflagellates in the French West Indies (Boisnoir et al. 2019, Chomérat et al. 2019). We also identified OTUs related to genus *Chrysochromulina* and *Prymnesium* in marine and mangrove waters, which correspond to reported toxic Prymnesiophyceae species (Houdan et al. 2004, Manning and La Claire 2010, Sobieraj and Metelski 2023, Wang et al. 2024). Finally, our molecular inventory of microbial taxa also indicated spatial heterogeneity in the Dinophyceae assemblage that will require more dedicated studies.

## Conclusion

The composition of the prokaryotic and eukaryotic microbial assemblages has rarely been investigated in Caribbean islands. Here, we provide a first description of both communities in three aquatic ecosystems: mangrove, marine, and urban environments around the most populated and anthropized area of Guadeloupe. This study provides detailed insights into the distribution of a large number of taxa and its heterogeneity. Sampling efforts were not uniform for the three compartments and information about physicochemical conditions would be required to obtain a full understanding of community assembly processes, but our findings show that turnover differed significantly between the three environments and was highest in urban areas, in which diversity was also greatest. This study reveals new molecular biomarkers for aquatic environments and detected, for the first time in this area, a number of OTUs potentially associated with toxic or harmful blooms. These baseline data will be very useful for efforts to monitor the impact of natural and anthropogenic pressures in Guadeloupe.

## Supplementary Material

fiag031_Supplemental_Files

## Data Availability

The raw data sets are available from the Sequence Read Archive under BioProject PRJNA1285926.
