# Supplementary material for: Patterns of microbial diversity in three aquatic ecosystems of a Caribbean island
Source: FEMS Microbiol Ecol. 2026 Mar 26;102(4):fiag031. doi: 10.1093/femsec/fiag031 (PMC13070568; doi:10.1093/femsec/fiag031)
Supplement: fiag031_Supplemental_Files [file fiag031_supplemental_files.zip › Supplementary_FigureS1.pdf]

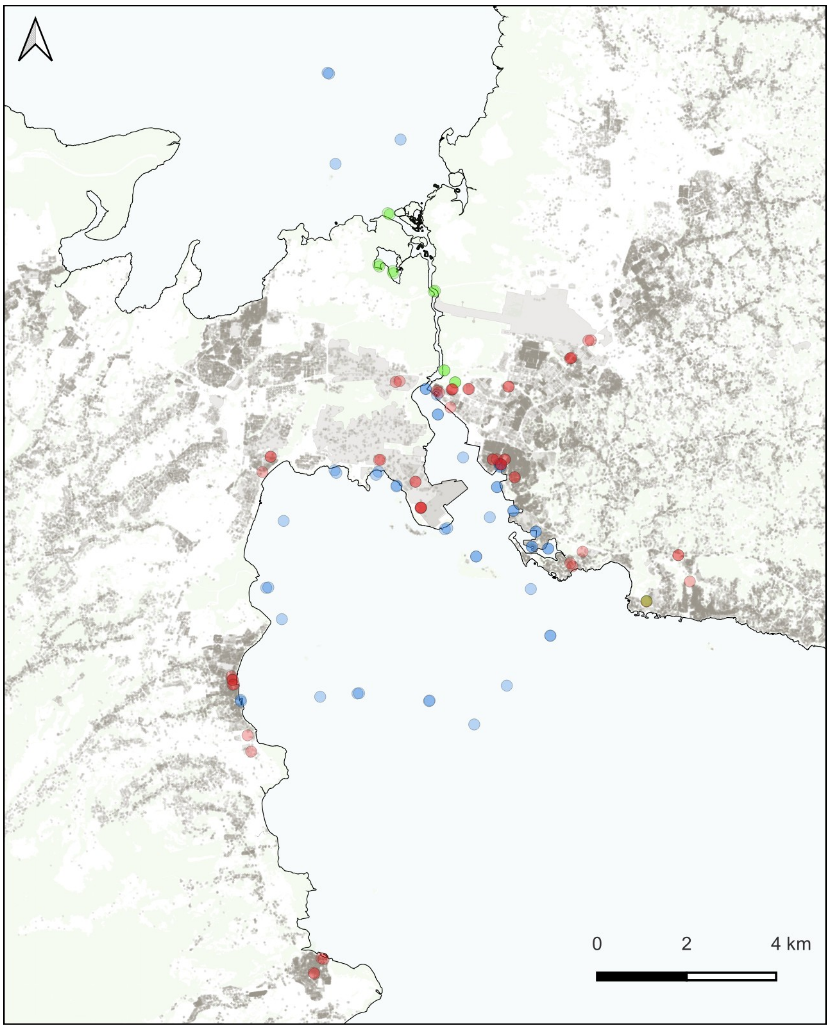

Supplementary Figure S1 | Map of the sampling location in Guadeloupe (French West Indies). Location of the sampling sites. The color code for the sampling sites corresponds to mangrove (in green), marine (blue) and urban (red). Note that the sampling sites can overlap; therefore, it can affect the color. The buildings, habitations and industrial areas are in light gray, and the vegetation in light green. BD Carto IGN, 2022.
