# Supplementary material for: Patterns of microbial diversity in three aquatic ecosystems of a Caribbean island
Source: FEMS Microbiol Ecol. 2026 Mar 26;102(4):fiag031. doi: 10.1093/femsec/fiag031 (PMC13070568; doi:10.1093/femsec/fiag031)
Supplement: fiag031_Supplemental_Files [file fiag031_supplemental_files.zip › Supplementary_FigureS14.pdf]

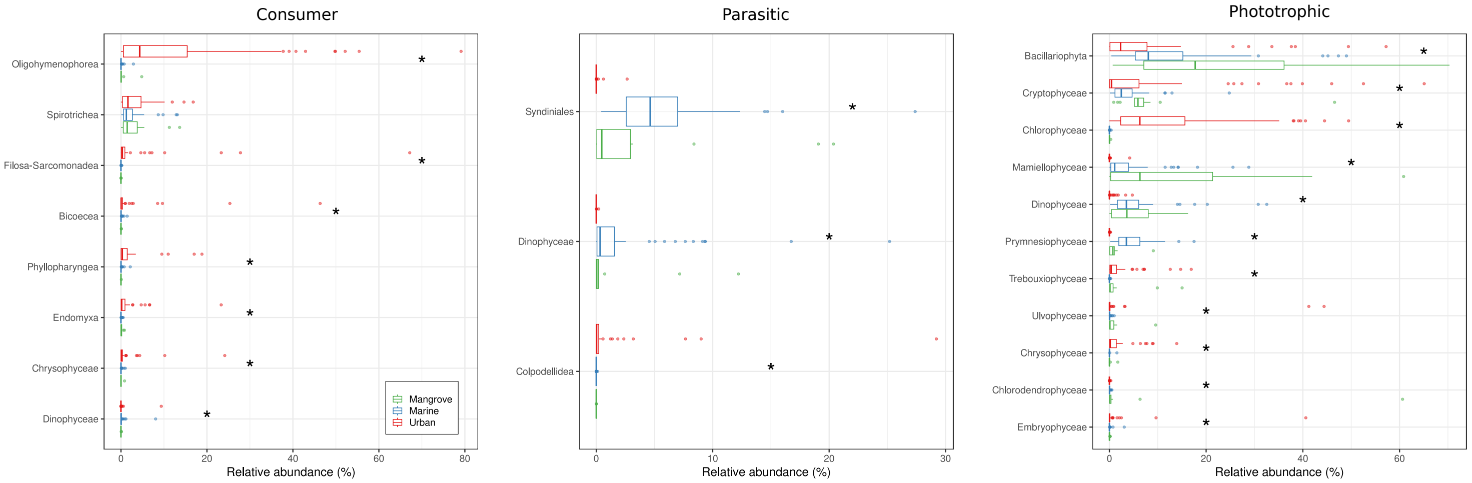

Supplementary Figure S14 | Boxplot showing the relative abundance of different classes of eukaryotes and according to their functional assignation. From left to right consumer, parasitic, and phototrophic. The asterisk corresponds to classes that present a differential abundance between the three environments using a Kruskal-Wallis test.
