# Supplementary material for: Patterns of microbial diversity in three aquatic ecosystems of a Caribbean island
Source: FEMS Microbiol Ecol. 2026 Mar 26;102(4):fiag031. doi: 10.1093/femsec/fiag031 (PMC13070568; doi:10.1093/femsec/fiag031)
Supplement: fiag031_Supplemental_Files [file fiag031_supplemental_files.zip › Supplementary_FigureS2.pdf]

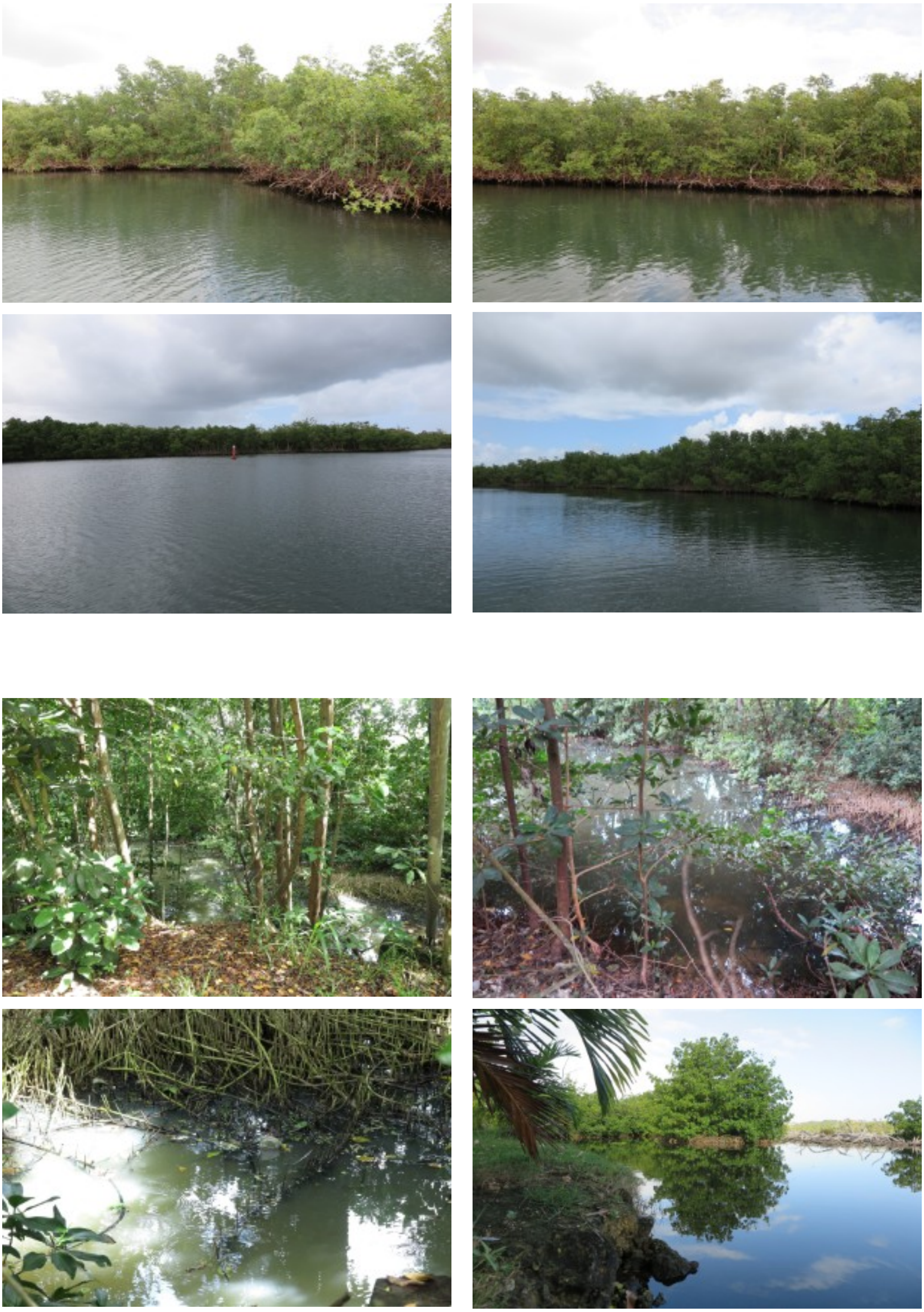

Supplementary Figure S2 | Photography from several mangrove sampling sites. The four upper images correspond to sampling sites along the mangrove fringe of the « rivière salée » and the « « manche à Eau ». The four lower images correspond to water sampling sites from inland mangrove forests.
