# Supplementary material for: Patterns of microbial diversity in three aquatic ecosystems of a Caribbean island
Source: FEMS Microbiol Ecol. 2026 Mar 26;102(4):fiag031. doi: 10.1093/femsec/fiag031 (PMC13070568; doi:10.1093/femsec/fiag031)
Supplement: fiag031_Supplemental_Files [file fiag031_supplemental_files.zip › Supplementary_FigureS4.pdf]

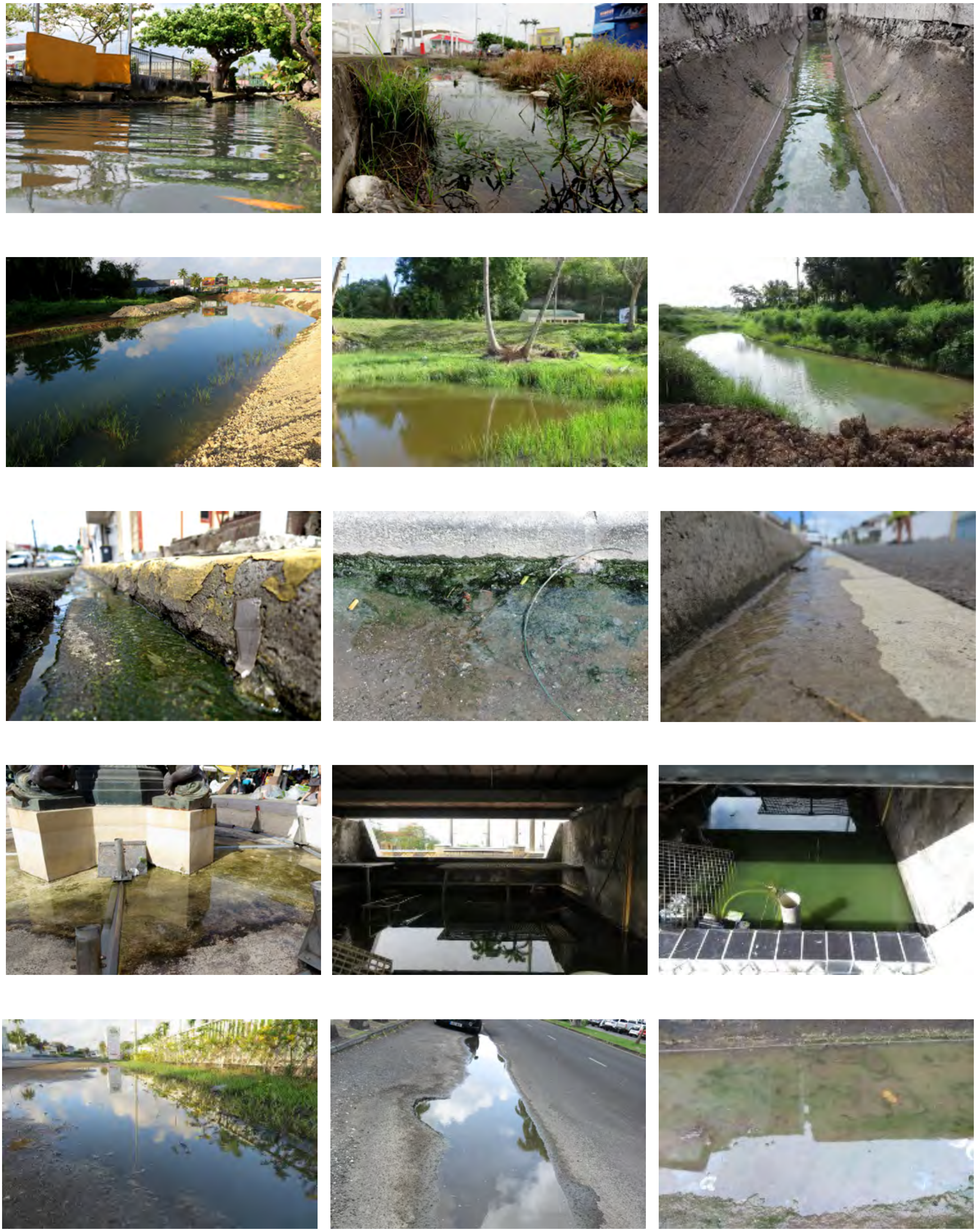

Supplementary Figure S4 | Photography from several urban sampling sites. From top to bottom, the pictures correspond to open pipeline network, artificial pond, street gutter, fountain, and episodic rain puddle.
