# Supplementary material for: Patterns of microbial diversity in three aquatic ecosystems of a Caribbean island
Source: FEMS Microbiol Ecol. 2026 Mar 26;102(4):fiag031. doi: 10.1093/femsec/fiag031 (PMC13070568; doi:10.1093/femsec/fiag031)
Supplement: fiag031_Supplemental_Files [file fiag031_supplemental_files.zip › Supplementary_FigureS5.pdf]

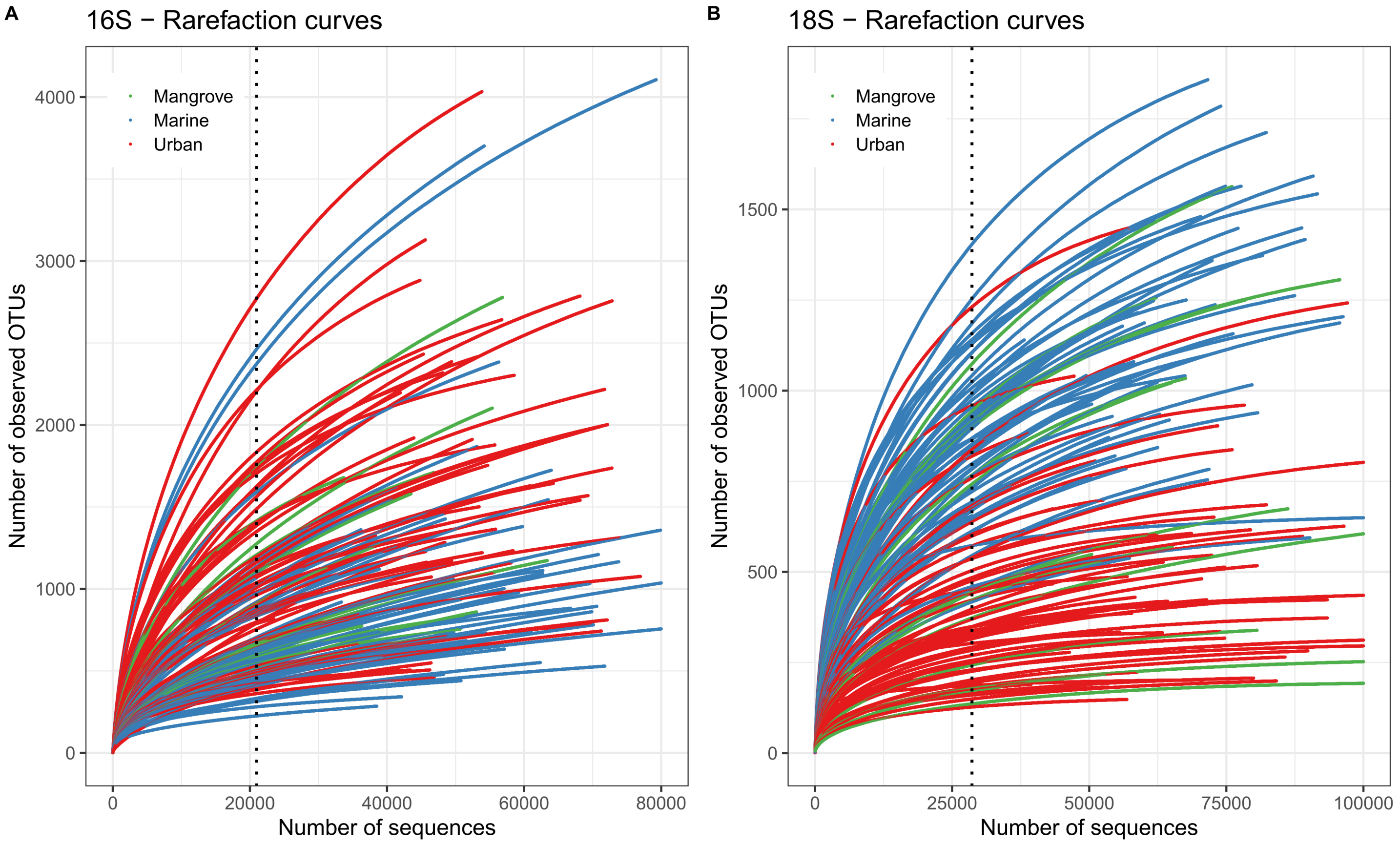


**Supplementary Supplementary Figure S5 | Rarefaction curves representing the number of OTUs against the number of high-quality reads.** (**A**) Prokaryotic OTUs and (**B**) eukaryotic OTUs.
