# Supplementary material for: Patterns of microbial diversity in three aquatic ecosystems of a Caribbean island
Source: FEMS Microbiol Ecol. 2026 Mar 26;102(4):fiag031. doi: 10.1093/femsec/fiag031 (PMC13070568; doi:10.1093/femsec/fiag031)
Supplement: fiag031_Supplemental_Files [file fiag031_supplemental_files.zip › Supplementary_FigureS6.pdf]

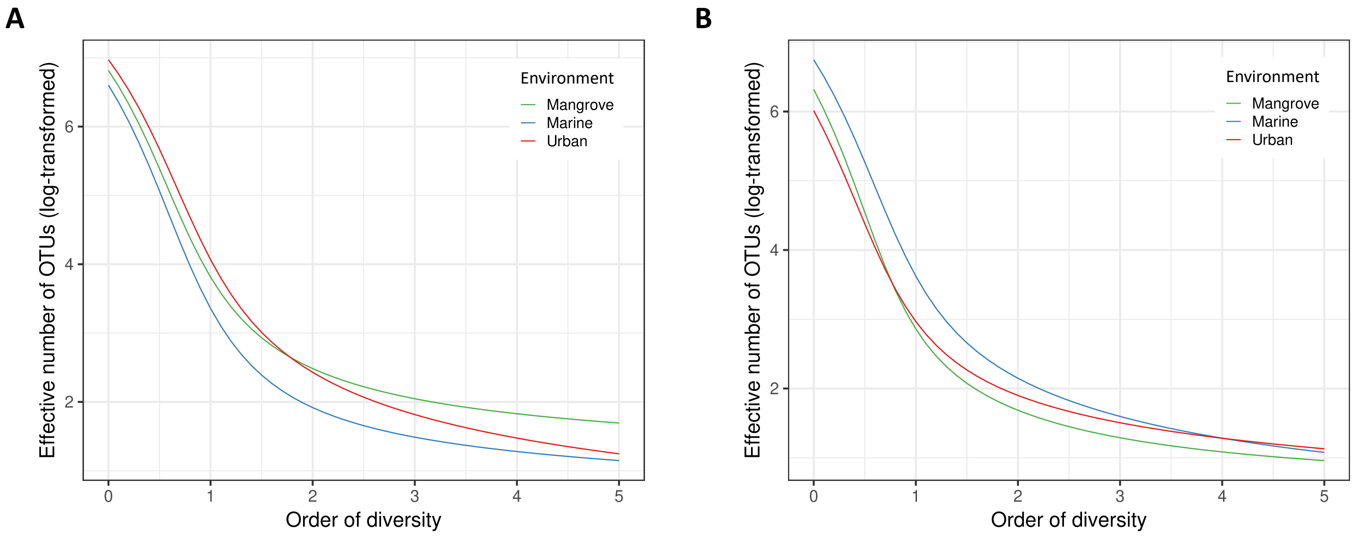

Supplementary Figure S6 | Alpha-diversity profile for prokaryotic and eukaryotic surface waters. (**A**) prokaryotes and (**B**) eukaryotes.
