# Supplementary material for: Patterns of microbial diversity in three aquatic ecosystems of a Caribbean island
Source: FEMS Microbiol Ecol. 2026 Mar 26;102(4):fiag031. doi: 10.1093/femsec/fiag031 (PMC13070568; doi:10.1093/femsec/fiag031)
Supplement: fiag031_Supplemental_Files [file fiag031_supplemental_files.zip › Supplementary_FigureS7.pdf]

A

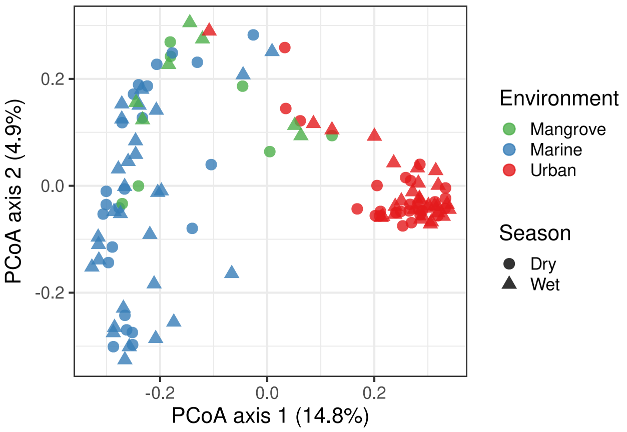


B

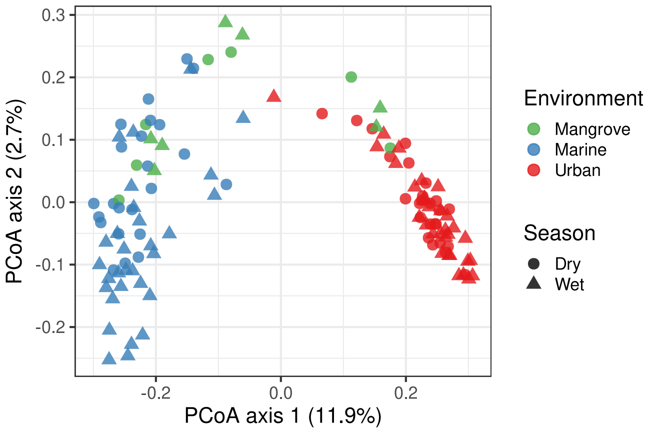


**Supplementary Figure S7 | Principal coordinate analyses (PCoA) of the microbial community composition based on Jaccard dissimilarity matrices showing differences in community composition between surface water samples.** (**A**) Prokaryotic and (**B**) eukaryotic communities.
