# Supplementary material for: Patterns of microbial diversity in three aquatic ecosystems of a Caribbean island
Source: FEMS Microbiol Ecol. 2026 Mar 26;102(4):fiag031. doi: 10.1093/femsec/fiag031 (PMC13070568; doi:10.1093/femsec/fiag031)
Supplement: fiag031_Supplemental_Files [file fiag031_supplemental_files.zip › Supplementary_FigureS8.pdf]

**A**

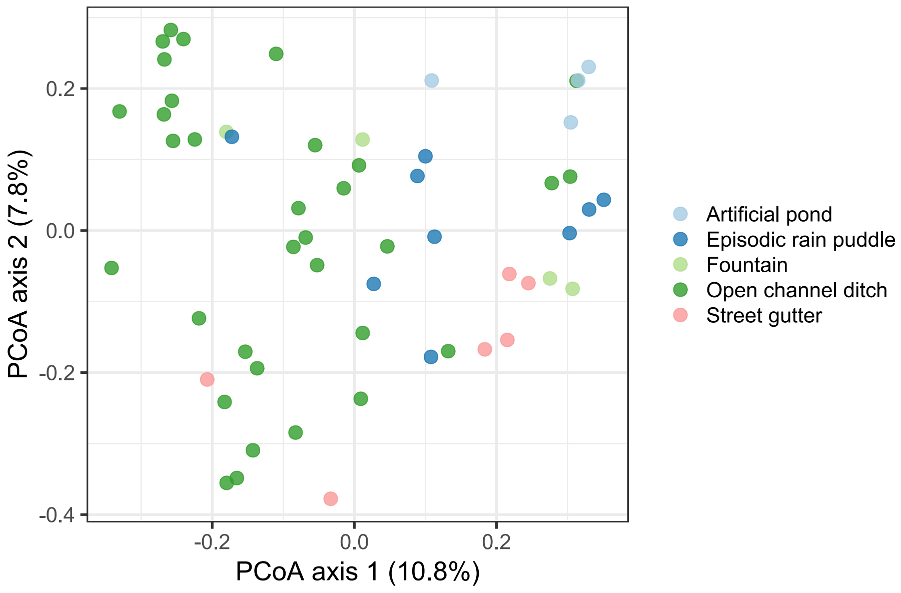


**B**

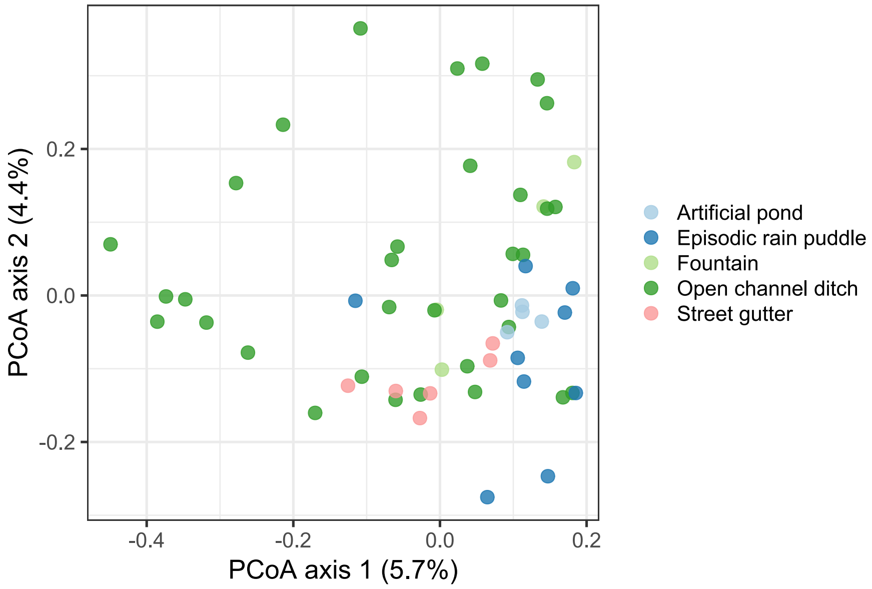


**Supplementary Figure S8 | Community composition in several urban sub compartments.** Principal coordinates analysis (PCoA) of the microbial community composition based on Bray-Curtis dissimilarity matrices in community composition between different urban waters sampled. (**A**) Prokaryotic community and (**B**) Eukaryotic community.
