# Supplementary material for: Patterns of microbial diversity in three aquatic ecosystems of a Caribbean island
Source: FEMS Microbiol Ecol. 2026 Mar 26;102(4):fiag031. doi: 10.1093/femsec/fiag031 (PMC13070568; doi:10.1093/femsec/fiag031)
Supplement: fiag031_Supplemental_Files [file fiag031_supplemental_files.zip › Supplementary_FigureS9.pdf]

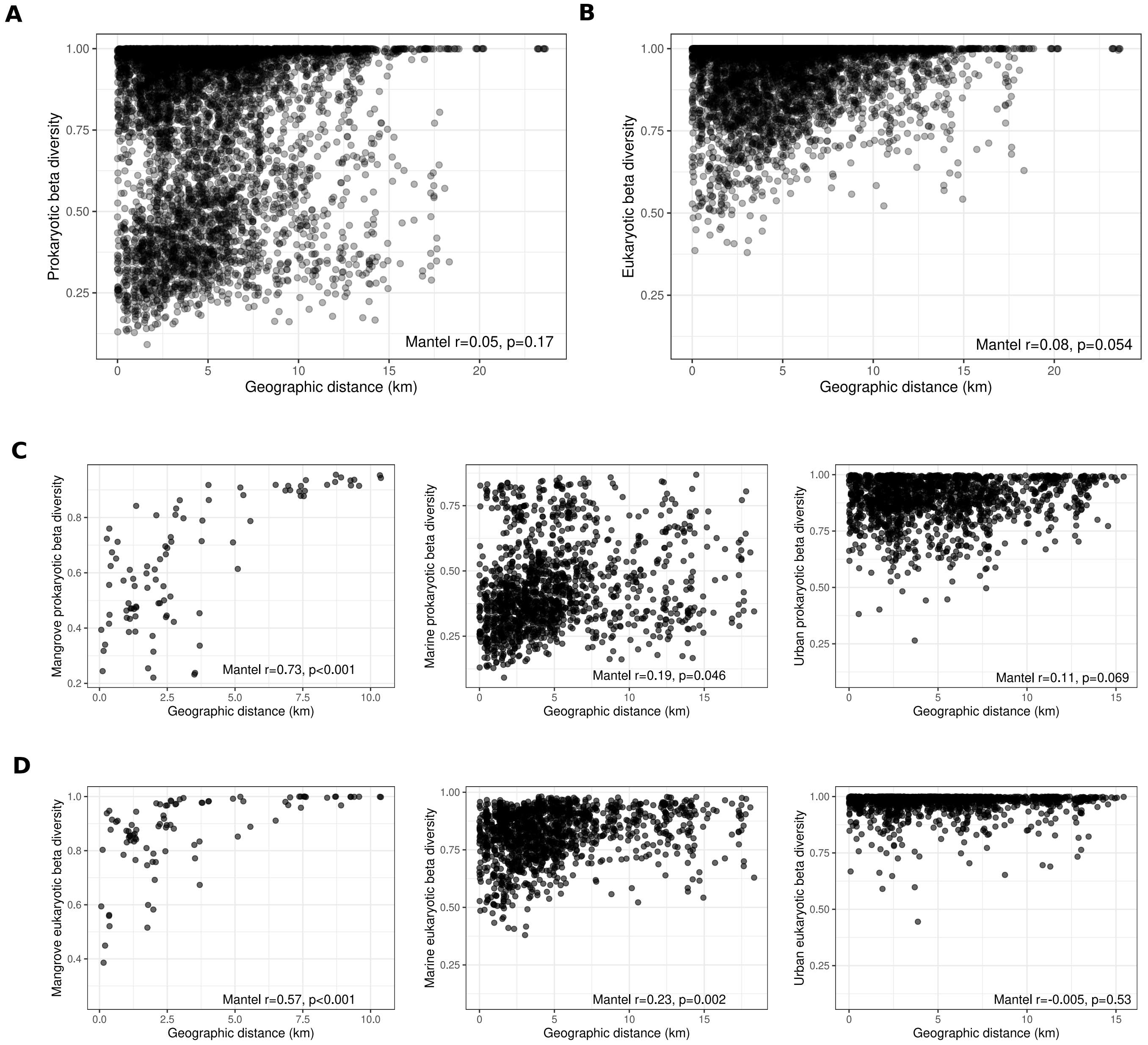

Supplementary Figure S9 | Relationship between spatial distance and community composition for the prokaryotes (A, C) and eukaryotes (B, D) for all datasets (A, B) and for each of the studied ecosystems (C, D). The correlation between the spatial and community matrices was assessed with a Mantel test.
