# Supplementary figures and images for: Patterns of microbial diversity in three aquatic ecosystems of a Caribbean island
Source: FEMS Microbiol Ecol. 2026 Mar 26;102(4):fiag031. doi: 10.1093/femsec/fiag031 (PMC13070568; doi:10.1093/femsec/fiag031)

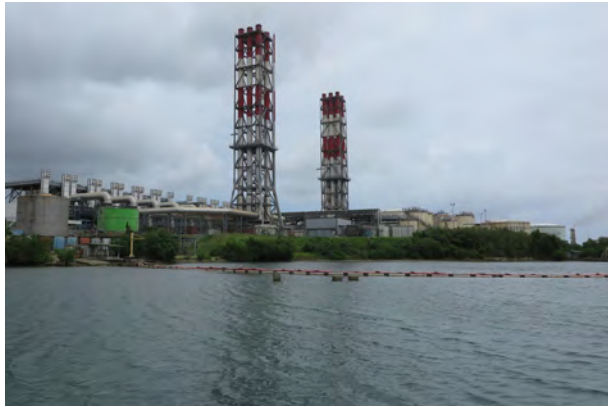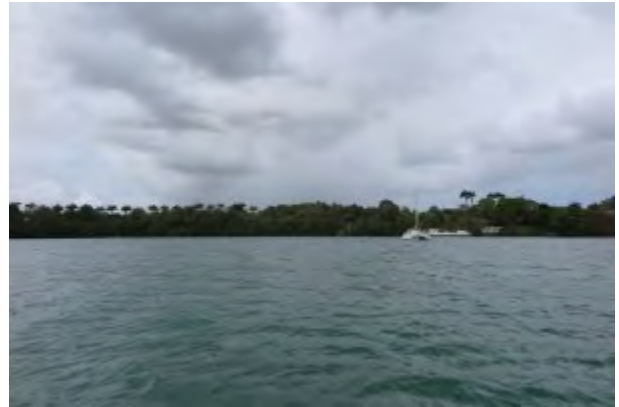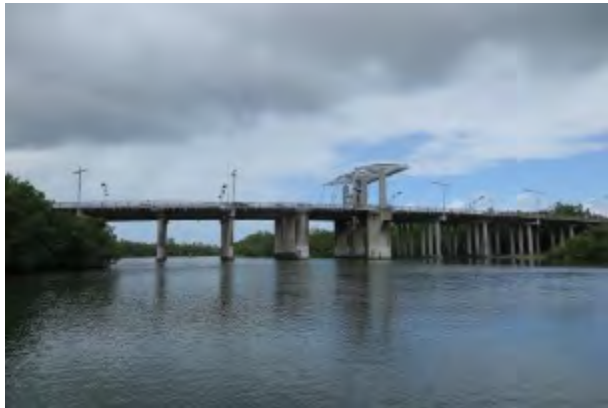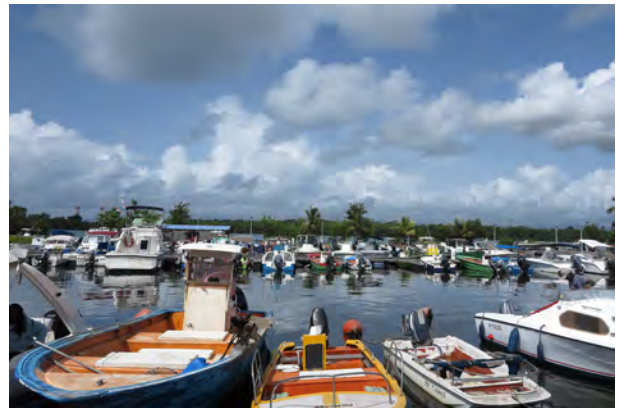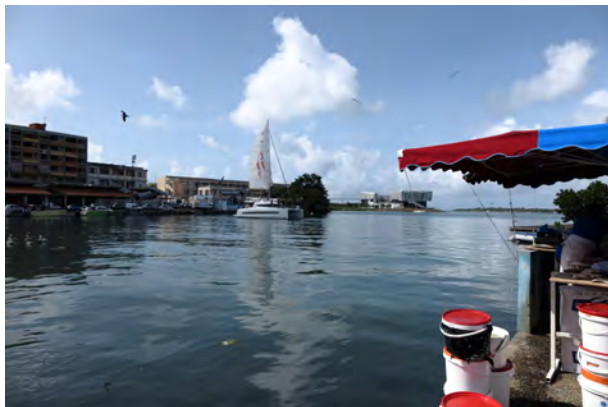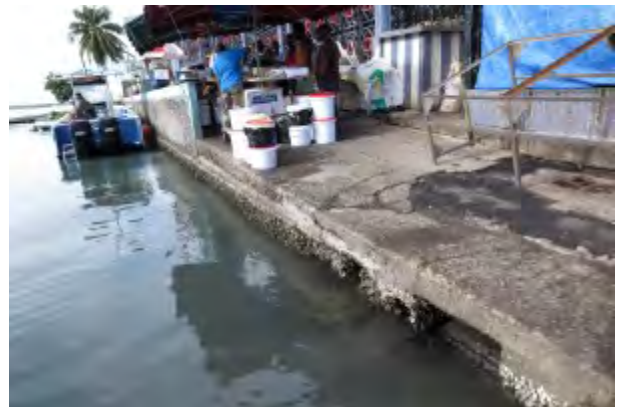

**Supplementary Figure S3 | Photography from several marine sampling sites.**

Supplement: fiag031_Supplemental_Files [file fiag031_supplemental_files.zip › Supplementary_FigureS3.pdf]
